# Syntheses and crystal structures of three tri­phenyl­sulfonium salts of manganese(II), iron(III) and cobalt(II)

**DOI:** 10.1107/S2056989025006668

**Published:** 2025-07-31

**Authors:** Waylan Callaway, Matthew Elterman, Nikita Krasilnikov, Gavin Roberts, Davis Rutan, Ty Spencer, Clifford W. Padgett, Will E. Lynch

**Affiliations:** a Center for Advanced Materials Science Department of Biochemistry Chemistry and Physics Georgia Southern University, 11935 Abercorn Street Savannah Georgia 31419 USA; Universidad de la Repüblica, Uruguay

**Keywords:** crystal structure, tri­phenyl­sulfonium ion, salts

## Abstract

Bis(tri­phenyl­sulfonium) tetra­chlorido­manganate(II), (C_18_H_15_S)_2_[MnCl_4_] (**I**), tri­phenyl­sulfonium tetra­chlorido­ferrate(III), (C_18_H_15_S)[FeCl_4_] (**II**), and bis­(tri­phenyl­sulfonium) tetra­chlorido­cobaltate(II), (C_18_H_15_S)_2_[CoCl_4_] (**III**), crystallize in the monoclinic space groups *P*2_1_/*n* [(**I**) and (**III**)] and *P*2_1_/*c* [(**II**)]. Compounds (**I**) and (**III**) each contain two crystallographically independent tri­phenyl­sulfonium (TPS^+^) cations in the asymmetric unit, whereas (**II**) has one. In all three compounds, the sulfonium centers adopt distorted trigonal–pyramidal geometries, with S—C bond lengths falling roughly in the 1.78–1.79 Å range and C—S—C angles observed at about 101 to 106°. The [*M*Cl_4_]^*n*−^ anions (*M* = Mn^2+^, Fe^3+^, Co^2+^; *n* = 2,1,2) adopt slightly distorted tetra­hedral geometries, with *M*—Cl bond lengths in the 2.19–2.38 Å range and Cl—*M*—Cl angles of approximately 104–113°. Hirshfeld surface analyses shows that H⋯H and H⋯C contacts dominate the TPS^+^ cation environments, whereas H⋯Cl and short *M*—S inter­actions link each [*M*Cl_4_]^*n*−^ anion to the surrounding cations. In (**I**) and (**III**), inversion-centered π–π stacking further consolidates the crystal packing, while in (**II**) no π–π inter­actions are observed.

## Abstract 2

The crystal structures of three salts of the tri­phenyl­sulfonium cation, C_18_H_15_S^+^, namely bis­(tri­phenyl­sulfonium) tetra­chlorido­manganate(II), tri­phenyl­sulfonium tetra­chlorido­ferrate(III) and bis­(tri­phenyl­sulfonium) tetra­chlorido­cobaltate(II) are reported.

## 1. Chemical context

A number of recent reports have put tri­phenyl­sulfonium (TPS^+^) salts in the spotlight due to their wide applications across various chemical processes. For example, a recent report (Imai, *et al.*, 2025 ▸) describes the development of new synthetic strategies to produce sterically demanding derivatives that improve the stability of the cation in basic environments. This enhanced stability is of inter­est to support anion-exchange membranes (AEMs) in both fuel cell and water-splitting technologies. These new TPS^+^ derivatives show promise in resisting degradation observed in other materials that are currently being used.

TPS^+^ salts are a subject of inter­est in photochemistry due to their role as photoacid generators (PAGs), producing acids in response to light exposure (Ohmori *et al.*, 1998 ▸). A previous report used tri­fluoro­methane­sulfonate tri­phenyl­sulfonium as a PAG to engineer potential photolinking resists (Lin *et al.*, 1997 ▸). This process has been significantly enhanced by adopting the tri­phenyl­sulfonium perfluoro-l-butane­sulfonate to produce high-resolution resist films, which are capable of being used in electron beam lithography (Zhang *et al.*, 2025 ▸). The photosensitive properties of these salts make them valuable in the production of computer chips and semiconductors (Kwon *et al.*, 2014 ▸; Wang *et al.*, 2023 ▸) and applications in anti-counterfeiting (Luo *et al.*, 2022*a* ▸).
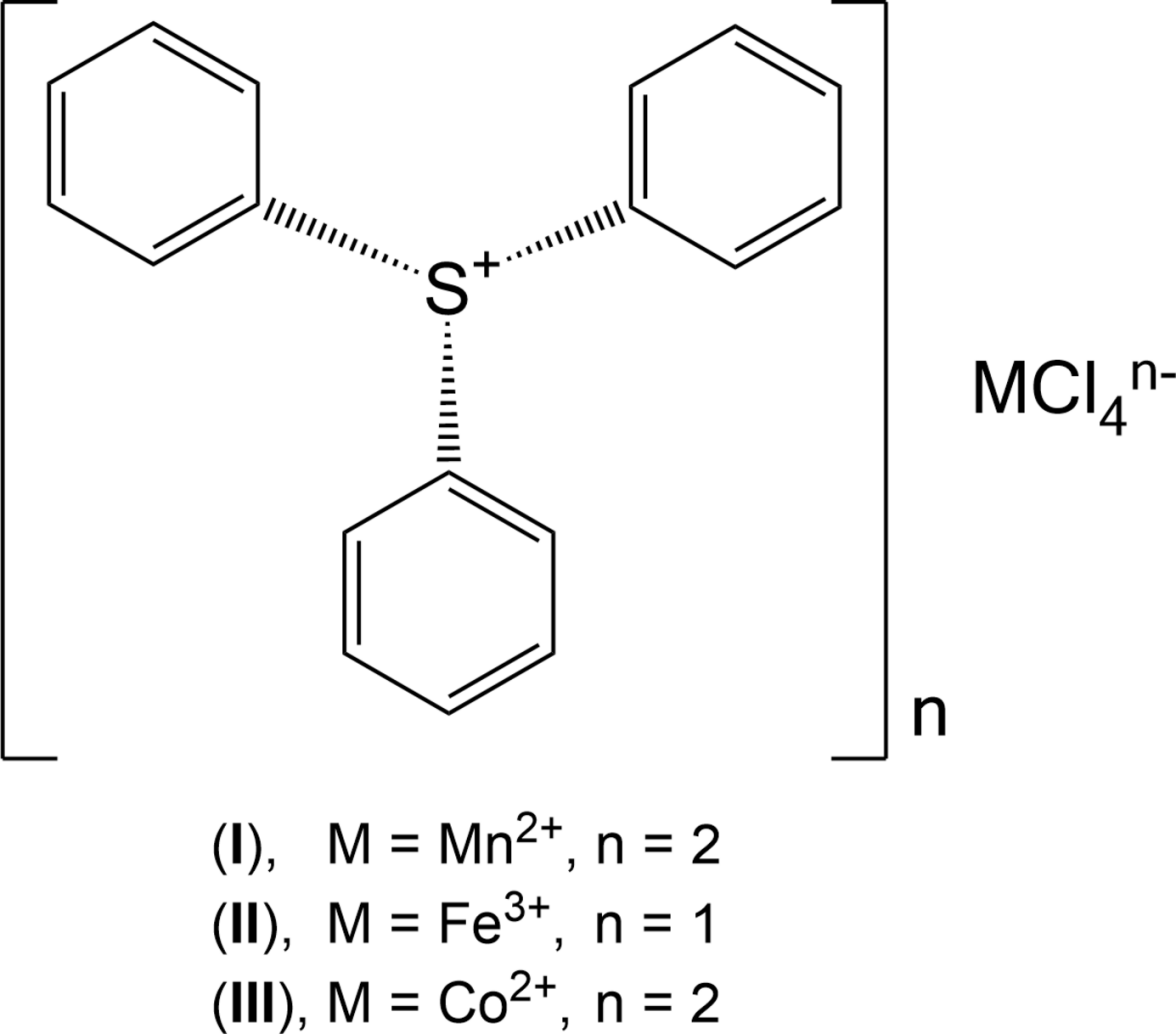


**Figure 1:**
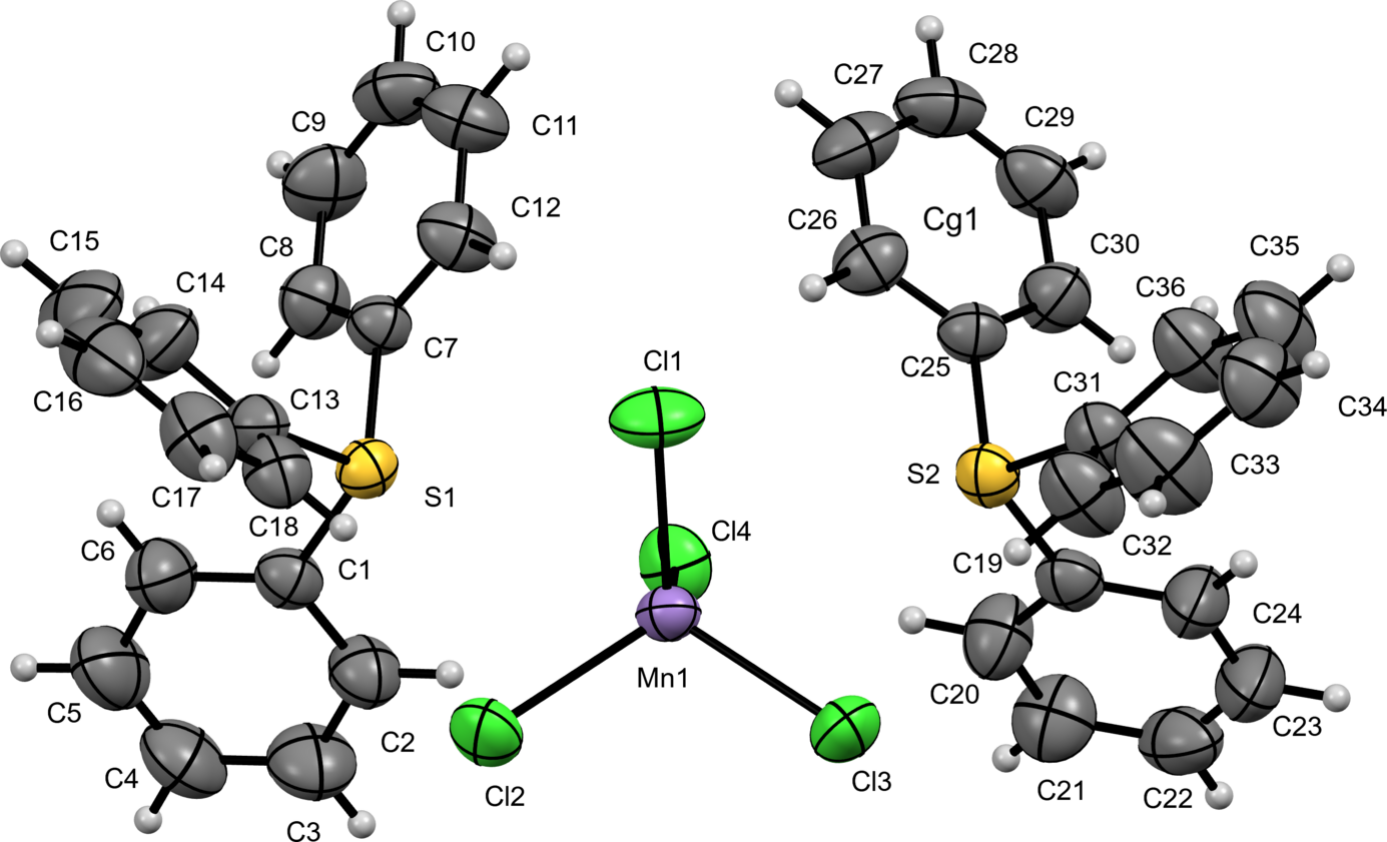
The mol­ecular structure of (**I**) with displacement ellipsoids drawn at the 50% probability level.

**Figure 2:**
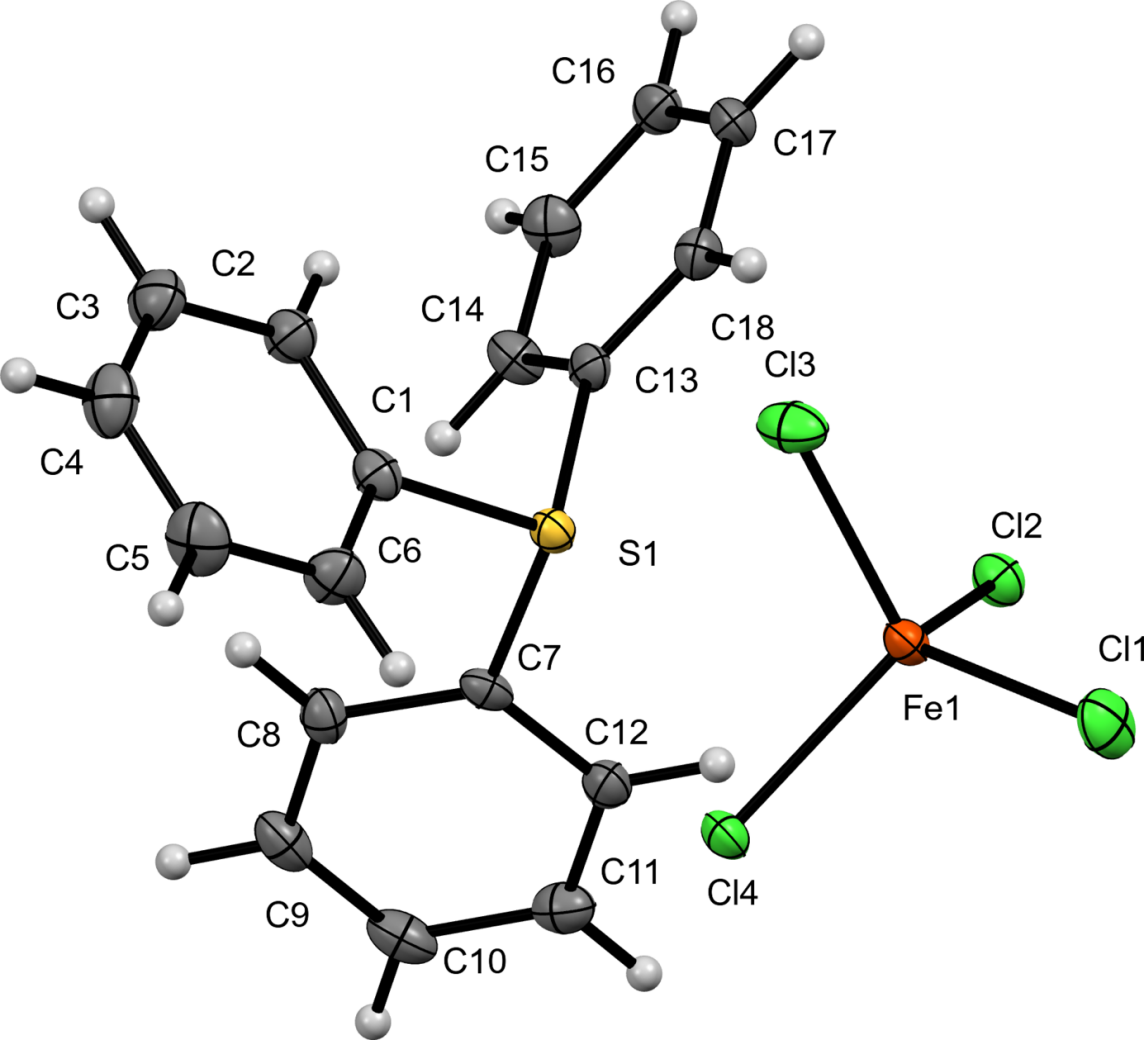
The mol­ecular structure of (**II**) with displacement ellipsoids drawn at the 50% probability level.

**Figure 3:**
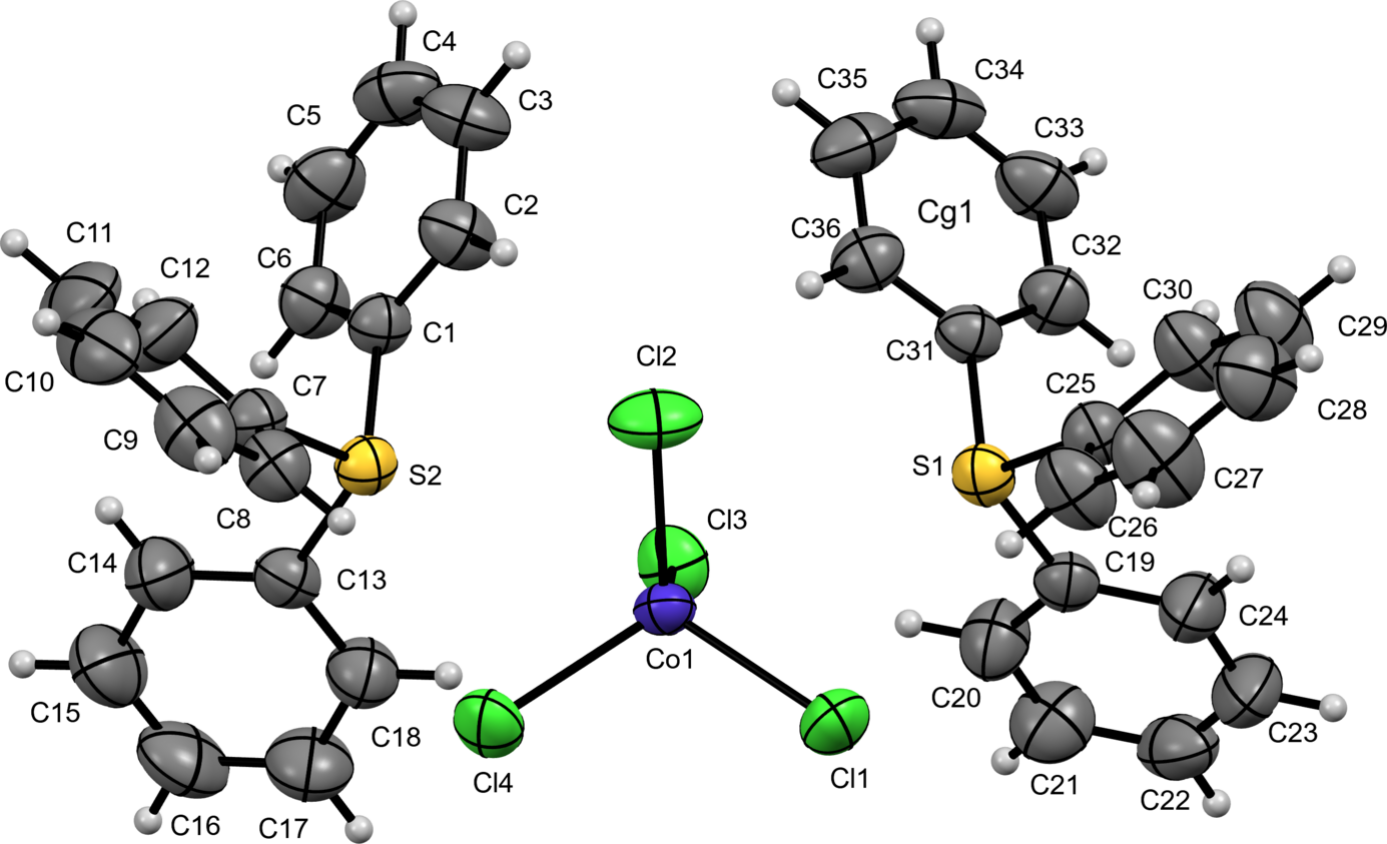
The mol­ecular structure of (**III**) with displacement ellipsoids drawn at the 50% probability level.

In this study, we report the crystal structures of three new TPS^+^ salts of first row transition-metal tetra­chlorido­metallate anions, namely: bis­(tri­phenyl­sulfonium) tetra­chlorido­mang­anate(II) (**I**) (Fig. 1 ▸), tri­phenyl­sulfonium tetra­chlorido­ferrate(III) (**II**) (Fig. 2 ▸), and bis­(tri­phenyl­sulfonium) tetra­chlorido­cobaltate(II) (**III**) (Fig. 3 ▸). These structures provide information regarding the importance of the metal halide complex ions into the packing, ionic inter­actions and properties of the tri­phenyl­sulfonium cation.

## 2. Structural commentary

Compound (**I**) crystallizes in the monoclinic space group *P*2_1_/*n*. The asymmetric unit of [TPS]_2_[MnCl_4_] comprises two crystallographically independent C_18_H_15_S^+^ tri­phenyl­sulfonium (TPS^+^) cations and one [MnCl_4_]^2−^ anion. Each sulfonium center exhibits a distorted trigonal–pyramidal geometry. In the first cation (containing S1), the S—C bond lengths range from 1.785 (5) to 1.793 (5) Å, while the C—S—C angles span 101.3 (2)–106.6 (2)°. In the second cation (containing S2), the S—C distances lie between 1.785 (5) and 1.791 (5) Å, and the C—S—C angles vary from 103.7 (2) to 105.2 (2)°. The [MnCl_4_]^2−^ anion adopts a slightly distorted tetra­hedral arrangement, with Mn—Cl bond lengths of 2.3421 (14)–2.3768 (14) Å and Cl—Mn—Cl angles in the 104.52 (6)–113.08 (6)° range.

Compound (**II**) crystallizes in the monoclinic space group *P*2_1_/c, with one crystallographically independent C_18_H_15_S^+^ tri­phenyl­sulfonium cation and one [FeCl_4_]^−^ anion in the asymmetric unit ([TPS][FeCl_4_]) . The sulfonium center (S1) exhibits a distorted trigonal–pyramidal geometry, with S—C bond lengths ranging from 1.781 (2) to 1.786 (2) Å, while the C—S—C angles vary from 103.90 (10) to 105.36 (10)°. The [FeCl_4_]^−^ anion adopts a slightly distorted tetra­hedral arrangement around Fe1, with Fe—Cl bond lengths of 2.1923 (6)—2.2020 (6) Å and Cl—Fe—Cl angles vary from 108.61 (2) to 110.23 (3)°.

Compound (**III**) crystallizes in the monoclinic space group *P*2_1_/*n*. The asymmetric unit of [TPS]_2_[CoCl_4_] comprises two crystallographically independent C_18_H_15_S^+^ tri­phenyl­sulfonium cations and one [CoCl_4_]^2−^ anion. Each sulfonium center exhibits a distorted trigonal–pyramidal geometry. In the first cation (containing S1), the S—C bond lengths ranging from 1.782 (2) to 1.791 (2) Å and C—S—C angles varying between 103.74 (10) to 104.97 (10)°. In the second cation (containing S2), the S—C distances lie between 1.787 (2) and 1.790 (2) Å, and the C—S—C angles span 101.57 (10)–106.37 (10)°. The [CoCl_4_]^2−^ anion adopts a slightly distorted tetra­hedral arrangement around Co1, with Co—Cl bond lengths of 2.2564 (7)–2.2893 (6) Å and Cl—Co—Cl angles ranging from 104.92 (3) to 112.61 (3)°.

## 3. Supra­molecular features

**Figure 4:**
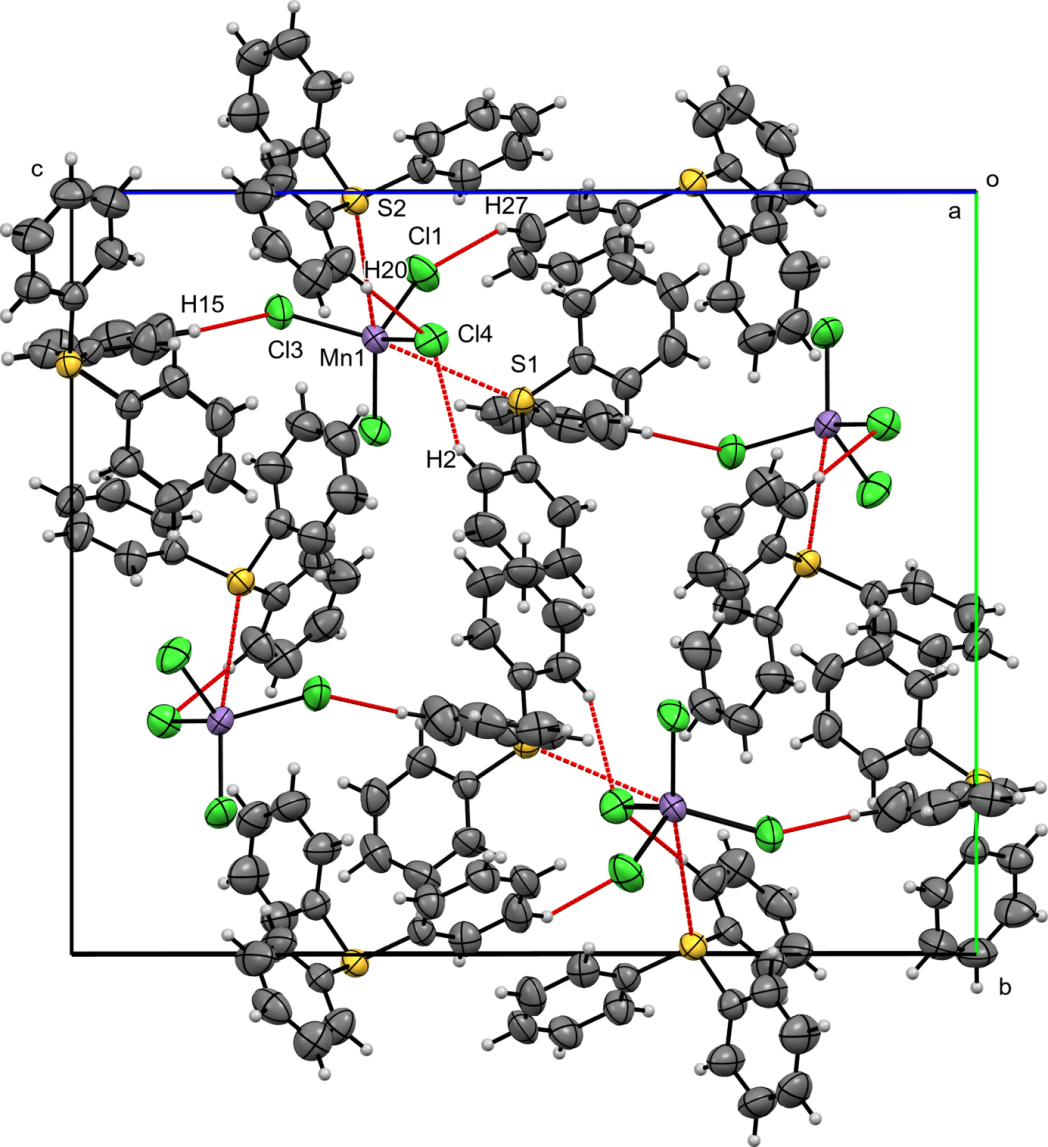
A view along the *a*-axis direction of the crystal packing of (**I**) with close contacts shown as red dashed lines.

Figs. 4 ▸, 5 ▸ and 6 ▸ illustrate the crystal packings of compounds (**I**), (**II**), and (**III**), respectively. In all three structures, the packing is consolidated by van der Waals and electrostatic inter­actions, and in compounds (**I**) and (**III**) π–π stacking is also observed. Hirshfeld surfaces were generated in *Crystal Explorer 21* (Spackman *et al.*, 2021 ▸) for each crystallographically independent tri­phenyl­sulfonium (TPS) cation and for the [*M*Cl_4_]^*n*−^ anion (*M* = Mn^2+^, Fe^3+^, Co^2+^; *n* = 2,1,2). The corresponding two-dimensional fingerprint plots (McKinnon *et al.*, 2007 ▸) were analyzed to qu­antify the relative contributions of the various inter­molecular contacts (Table 1 ▸). Hydrogen bonds for (**I**), (**II**) and (**III**) are listed in Tables 2 ▸–4 ▸ ▸, respectively.

In the crystal structure of compound (**I**), two TPS^+^ cations (TPS1 and TPS2) occur in the asymmetric unit. On the Hirshfeld surfaces of TPS1 and TPS2, H⋯H inter­actions dominate, accounting for 49.8% (TPS1) and 54.5% (TPS2), followed by H⋯C contacts at 30.0% (TPS1) and 20.7% (TPS2). The C⋯C contacts are minor (3.8% for TPS1; 5.5% for TPS2). Notably, H⋯Cl contacts (14.3% for TPS1; 16.1% for TPS2) reflect hydrogen-bond-like inter­actions with the [MnCl_4_]^2–^ anion. The [MnCl_4_]^2–^ Hirshfeld surface is dominated by H⋯Cl (90.2%), with S⋯Cl (4.3%) and S⋯Mn (1.5%) also present (Table 1 ▸). Discrete (TPS)_2_–MnCl_4_ units are formed through Mn1—S1 and Mn1—S2 short contacts at 3.7548 (13) and 3.8243 (14) Å, respectively, along with C—H⋯Cl inter­actions [H2⋯Cl4 = 2.6828 (14), H20⋯Cl4 = 2.7038 (14) Å]. These units are linked into chains *via* Cl3—H15 [2.6834 (16) Å; symmetry code: 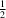 + *x*, 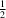 − *y*, −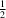 + *z*] parallel to the (101) plane, thus forming extended layers. These C—H⋯Cl short contacts can be regarded as weak hydrogen bonds (Steiner *et al.*, 1998 ▸). A single inversion-centered π–π stacking inter­action [*Cg*1⋯*Cg*1^i^, symmetry code: (i) 2 − *x*, −*y*, 1 − *z*); centroid–centroid separation = 3.807 (5) Å, shift = 1.520 (9) Å; *Cg*1 is the centroid of the C25–C30 ring].

In the crystal structure of compound (**II**), one independent TPS^+^ cation (TPS1) occurs in the asymmetric unit. H⋯H inter­actions dominate, occupying 42.4% of the Hirshfeld surface, followed by H⋯C at 19.6%, with minor C⋯C contacts, 0.4%. Hydrogen-bond-like inter­actions with [FeCl_4_]^−^ appear as H⋯Cl contributions of 27.9% of the surface. On the anion Hirshfeld surface, H⋯Cl inter­actions dominate (81.0%), with S⋯Cl (2.9%) and S⋯Fe (0.7%) also being observed. TPS–FeCl_4_ units are held together by short Fe—S [Fe1—S1 = 3.7092 (6) Å]. Additional short contacts include H16—Cl4 [2.7926 (5) Å; symmetry code: 1 − *x*, 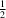 + *y*, 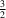 − *z*), H15—Cl1 [2.7527 (7) Å; symmetry code: −1 + *x*, 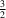 − *y*, 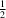 + *z*], and Fe—C [C11—Fe1 = 3.905 (3), C17—Fe1 = 3.896 (2) Å] contacts. These contacts generate a di-periodic layer parallel to the (102) plane. No π–π stacking inter­actions are observed.

In the crystal structure of compound (**III**), as in (**I**), two independent TPS^+^ cations (TPS1, TPS2) occur in the asymmetric unit. H⋯H inter­actions dominate (54.6% for TPS1; 50.1% for TPS2) the Hirshfeld surface, followed by H⋯C (20.9% for TPS1; 30.1% for TPS2), with minor C⋯C contacts (5.6% for TPS1; 3.8% for TPS2). Hydrogen-bond-like inter­actions with [CoCl_4_]^2–^ appear as H⋯Cl contributions of 15.9% (TPS1) and 13.9% (TPS2). On the anion Hirshfeld surface, H⋯Cl inter­actions dominate (90.4%), with S⋯Cl (4.7%) and S⋯Co (1.5%) also being observed. As seen with compound (**I**), discrete (TPS)_2_–CoCl_4_ units are observed and are formed by short Co—S contacts, Co1—S1 at 3.7873 (7) Å and Co1—S2 at 3.7183 (6) Å, as well as C—H⋯Cl inter­actions involving Cl3 [H18⋯Cl3 = 2.6789 (6), H20⋯Cl3 = 2.7031 (6) Å]. These units are extended into chains in the (101) plane by Cl1—H11 [2.7007 (7) Å; symmetry code: 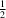 + *x*, 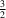 − *y*, −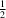 + *z*]. A single inversion-centered π–π stacking inter­action is present [*Cg*1··*·Cg*1^i^, symmetry code: (i) −*x*, 1 − *y*, 1 − *z*); centroid–centroid separation = 3.794 (2) Å, shift = 1.476 (4) Å; *Cg*1 is the centroid of the C31–C36 ring].

## 4. Database survey

A search of the web-based Cambridge Structural Database (CSD; website, accessed on June 3, 2025; Groom *et al.*, 2016 ▸) for the tri­phenyl­sulfonium ion yielded 27 entries with 22 being TPS^+^ complexes. Of the five reported structures that were not tri­phenyl­sulfonium ions, two were imine derivatives, one was a thia­zine motif and two are nitrile derivatives of tri­phenyl­sulfonium.

Related tetra­chlorido­metallate(II) salts recently reported from our lab include the zinc(II) (KUSQIC; Artis *et al.*, 2025*b* ▸), cadmium(II) (KUSQOI; Artis *et al.*, 2025*b* ▸) and mercury(II) (KUSQUO; Artis *et al.*, 2025*b* ▸). Further, we have also recently published the triiodide (FUMMEJ; Artis *et al.*, 2025*a* ▸), perchlorate (FUMMIN; Artis *et al.*, 2025*a* ▸) and hexa­fluoro­phosphate (FUMMOT; Artis *et al.*, 2025*a* ▸) salts.

Previous, simple salts derivatives of TPS^+^ include the bis­[(tri­fluoro­meth­yl)sulfon­yl]aza­dine salt (CSD refcode BANYOH; Siu *et al.*, 2017 ▸), azide (FOYKEK; Klapötke *et al.*, 2009*a* ▸), tri­fluoro­methansulfonate (LECWOI; Zhang *et al.*, 2017 ▸), chloride monohydrate (NIMMIJ; Luo *et al.*, 2022*b* ▸), bromide hydrate (ROKYAS; Klapötke *et al.*, 2009*a* ▸), tetra­fluoro­borate (TUBXET; Ovchinnikov *et al.*, 1996 ▸).

Metal-based anionic salts of anti­mony, tin and tellurium of the formula [TPS]_2_*M*Cl_*x*_ (where *X* = 5 or 6) include the bis­(tri­phenyl­sulfonium) penta­chloro­anti­monate(III) (MUFFAY; Liao *et al.* 2024 ▸) and its aceto­nitrile solvate (MUFFIG; Liao *et al.* 2024 ▸), the bis­(tri­phenyl­sulfonium) hexa­chloro­stannate(IV) (NIMMAB; Luo *et al.*, 2022*b* ▸), and bis­(tri­phenyl­sulfonium) hexa­chloro­tellurate(V) (NIMMEF; Luo *et al.*, 2022*b* ▸).

More unique structures are reported including the bis­(μ^2^-1,3-azido)­silver(I) (QOSQEV; Klapötke *et al.*, 2009*b* ▸) and the tris­(μ^2^-dicyanamido)­manganese(II) (SABFUX; Schlueter, *et al.*, 2004 ▸) structures with tri­phenyl­sulfonium. Two tris­(penta­fluoro­phen­yl)borate structures have been reported, [MIHKER (Khalimon *et al.*, 2012 ▸) and WUTBIY (Khalimon *et al.*, 2015 ▸)] and a bromide salt with 1,3,5-tri­fluoro-2,4,6-tris­(iodo­ethyn­yl)benzene (IFAMUZ; Lieffrig, *et al.*, 2013 ▸).

## 5. Synthesis and crystallization

Bis(tri­phenyl­sulfonium) tetra­chlorido­manganate(II), [C_18_H_15_S]_2_[MnCl_4_], compound (**I**) was synthesized by reacting 0.100 g (0.335 mmol) of tri­phenyl­sulfonium chloride in 5 mL of methanol in a 50 mL beaker. Separately, 0.0330 g of MnCl_2_^.^4H_2_O (0.167 mmol) were dissolved similarly in 5 mL of methanol, and the solutions were mixed with stirring for 10 minutes. Crystals were grown at 295 K by slow evaporation over one week resulting in tan , X-ray quality crystals that were isolated *via* vacuum filtration. Yield, 0.0501 g (41.5%). Selected IR bands (ATR-IR, cm^−1^): 1478 (*w*), 1444 (*w*), 1063 (*w*), 997 (*w*), 744 (*w*), 680 (*w*), 495 (*w*).

Tri­phenyl­sulfonium tetra­chlorido­ferrate(III), [C_18_H_15_S][FeCl_4_], compound (**II**), was synthesized by dissolving 0.0878 g FeCl_3_^.^6H_2_O (0.325 mmol) in 3 mL of methanol. To this solution, 3 mL of a 0.111 *M* tri­phenyl­sulfonium chloride methanol solution were added at 295 K. The subsequent solution was stirred for 10 minutes and then covered with a watch glass. X-ray quality crystals were grown by slow evaporation at 295 K and isolated by vacuum filtration. Yield, 0.1362 g (90.9%). IR bands (ATR-IR, cm^−1^): 1475 (*m*), 1446 (*m*), 1311 (*w*), 996 (*w*), 750 (*s*), 740 (*s*), 680 (*s*), 495 (*s*).

Bis(tri­phenyl­sulfonium) tetra­chlorido­cobaltate(II), compound (**III**) was synthesized by dissolving of 0.1016 g of tri­phenyl­sulfonium chloride (0.340 mmol) in 5 mL of methanol. To this solution was added 0.0404 g of CoCl_2_^.^6H_2_O (0.170 mmol) in one portion. The solution was stirred to dissolve the cobalt(II) chloride and the resulting solution was covered with Parafilm and allowed to evaporate for one week at 295 K. The product was isolated *via* vacuum filtration and the final mass was 0.0702 g (54.9%). Selected IR bands (ATR-IR, cm^−1^): 1737.28(*s*), 1477.34(*s*), 1444.85(*s*), 1065.10(*s*), 995.21(*s*), 747.55(*s*), 682.37(*s*), 499.73(*s*).

## 6. Refinement

Crystal data, data collection and structure refinement details are summarized in Table 5 ▸. All carbon-bound H atoms were positioned geometrically and refined as riding atoms: C—H = 0.95–0.98 Å with *U*_iso_(H) = 1.2*U*_eq_(C).

## Supplementary Material

Crystal structure: contains datablock(s) I, II, III. DOI: 10.1107/S2056989025006668/ny2014sup1.cif

Structure factors: contains datablock(s) I. DOI: 10.1107/S2056989025006668/ny2014Isup2.hkl

Structure factors: contains datablock(s) II. DOI: 10.1107/S2056989025006668/ny2014IIsup3.hkl

Structure factors: contains datablock(s) III. DOI: 10.1107/S2056989025006668/ny2014IIIsup4.hkl

CCDC references: 2475738, 2475737, 2475736

Additional supporting information:  crystallographic information; 3D view; checkCIF report

## Figures and Tables

**Figure 5:**
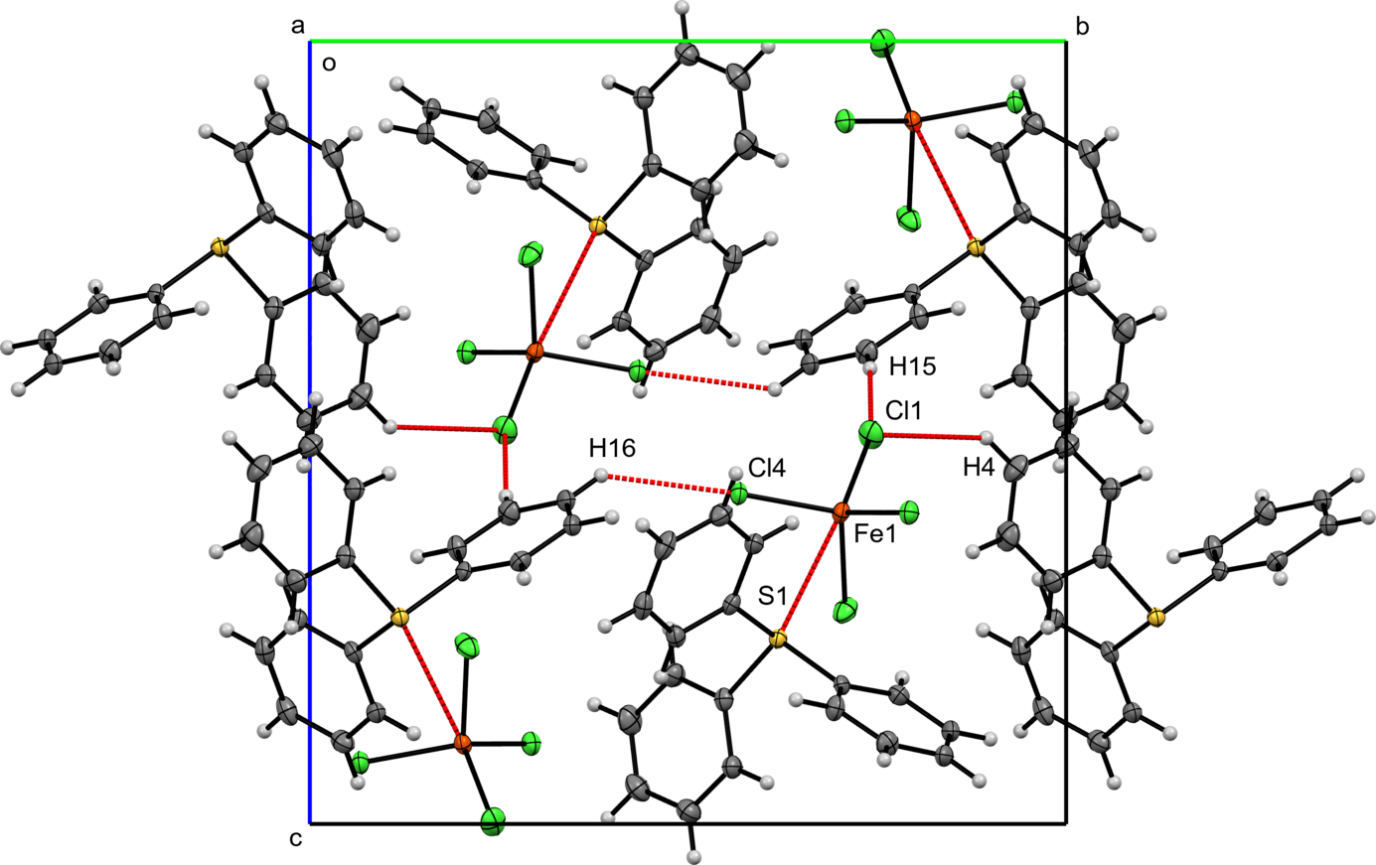
A view along the *a*-axis direction of the crystal packing of (**II**) with close contacts shown as red dashed lines.

**Figure 6:**
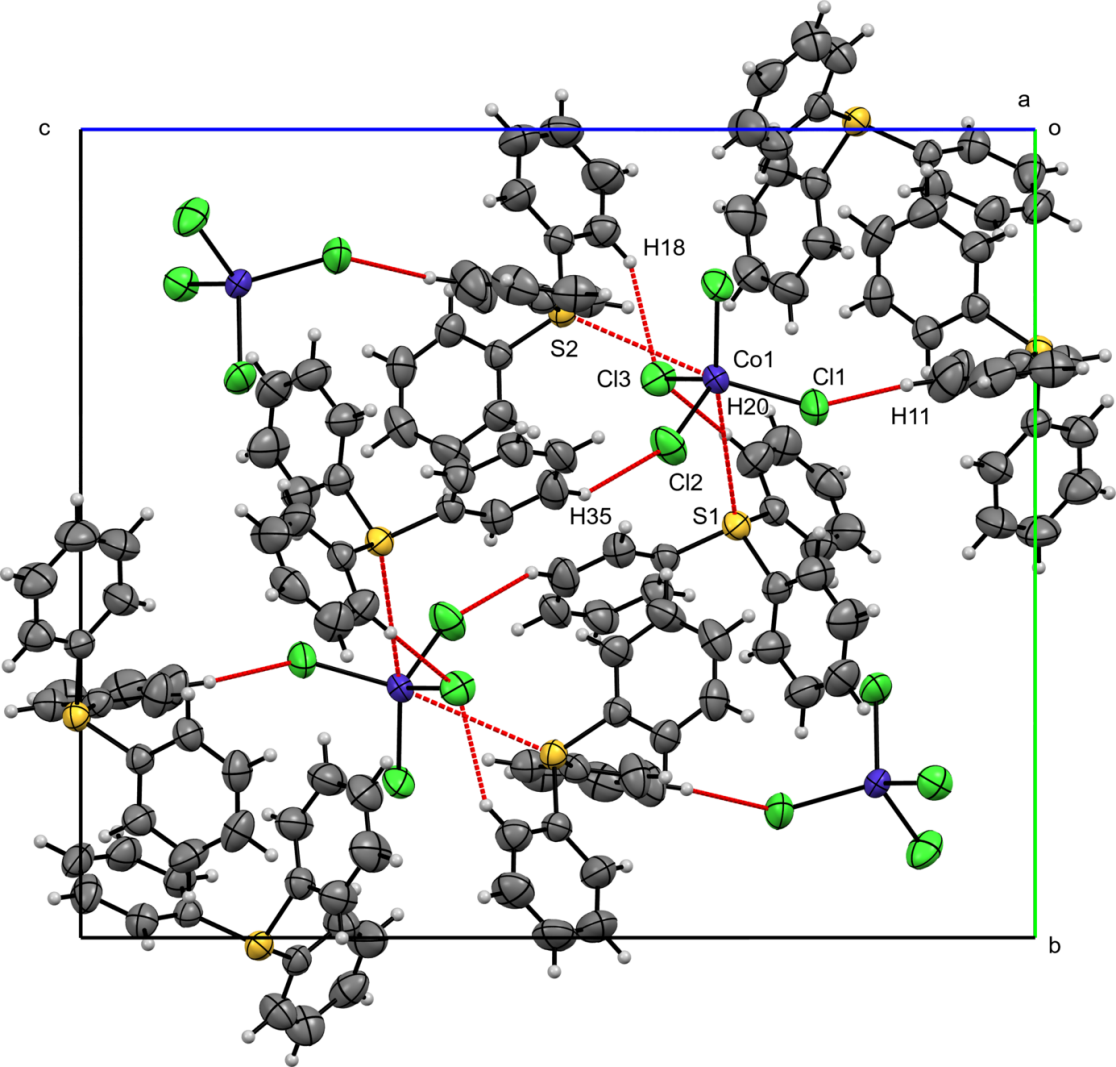
A view along the *a*-axis direction of the crystal packing of (**III**) with close contacts shown as red dashed lines.

**Table 1:** Contributions of selected inter­molecular contacts (%)

Contact	(**I**) (TPS1)	(**I**) (TPS2)	(**I**) (MnCl_4_)	(**II**) (TPS1)	(**II**) (FeCl_4_)	(**III**) (TPS1)	(**III**) (TPS2)	(**III**) (CoCl_4_)
C⋯C	3.8	5.5	–	0.4	–	5.6	3.8	–
H⋯C	30.0	20.7	–	19.6	–	20.9	30.1	–
H⋯H	49.8	54.5	–	42.4	–	54.6	50.1	–
H⋯Cl	14.3	16.1	90.2	27.9	81.0	15.9	13.9	90.4
S⋯Cl	1.2	1.1	4.3	1.5	2.9	1.2	1.3	4.7
S⋯*M*	0.4	0.4	1.5	0.4	0.7	0.4	0.4	1.5

**Table 2:** Hydrogen-bond geometry (Å, °) for (**I**)

*D*—H⋯*A*	*D*—H	H⋯*A*	*D*⋯*A*	*D*—H⋯*A*
C2—H2⋯Cl4	0.93	2.68	3.530 (6)	152
C15—H15⋯Cl3^i^	0.93	2.68	3.557 (6)	157
C20—H20⋯Cl4^ii^	0.93	2.70	3.589 (6)	159
C27—H27⋯Cl1	0.93	2.72	3.571 (6)	152

Symmetry codes: (i) 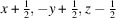; (ii) 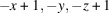.

**Table 3:** Hydrogen-bond geometry (Å, °) for (**II**)

*D*—H⋯*A*	*D*—H	H⋯*A*	*D*⋯*A*	*D*—H⋯*A*
C4—H4⋯Cl1^i^	0.95	2.89	3.676 (3)	141
C15—H15⋯Cl1^ii^	0.95	2.75	3.693 (2)	171
C16—H16⋯Cl4^iii^	0.95	2.79	3.489 (2)	131

Symmetry codes: (i) 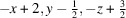; (ii) 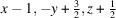; (iii) 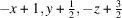.

**Table 4:** Hydrogen-bond geometry (Å, °) for (**III**)

*D*—H⋯*A*	*D*—H	H⋯*A*	*D*⋯*A*	*D*—H⋯*A*
C11—H11⋯Cl1^i^	0.93	2.70	3.581 (3)	158
C18—H18⋯Cl3	0.93	2.68	3.525 (3)	152
C20—H20⋯Cl3	0.93	2.70	3.582 (3)	158
C35—H35⋯Cl2^ii^	0.93	2.74	3.585 (3)	151

Symmetry codes: (i) 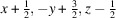; (ii) 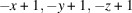.

**Table 5:** Experimental details

	(**I**)	(**II**)	(**III**)
Crystal data			
Chemical formula	(C_18_H_15_S)_2_[MnCl_4_]	(C_18_H_15_S)[FeCl_4_]	(C_18_H_15_S)[CoCl_4_]
*M*_r_	723.46	461.01	727.45
Crystal system, space group	Monoclinic, *P*2_1_/*n*	Monoclinic, *P*2_1_/*c*	Monoclinic, *P*2_1_/*n*
Temperature (K)	297	100	297
*a*, *b*, *c* (Å)	9.3550 (4), 17.7628 (8), 21.3952 (9)	8.3336 (1), 15.2143 (2), 16.0128 (2)	9.3200 (2), 17.7236 (3), 21.2341 (3)
β (°)	99.465 (4)	100.175 (1)	99.331 (2)
*V* (Å^3^)	3506.9 (3)	1998.33 (4)	3461.12 (11)
*Z*	4	4	4
Radiation type	Cu *K*α	Cu *K*α	Cu *K*α
μ (mm^−1^)	7.16	11.92	8.04
Crystal size (mm)	0.22 × 0.14 × 0.06	0.18 × 0.15 × 0.10	0.21 × 0.17 × 0.17

Data collection			
Diffractometer	XtaLAB Synergy, Single source at home/near, HyPix3000	XtaLAB Synergy, Single source at home/near, HyPix3000	XtaLAB Synergy, Single source at home/near, HyPix3000
Absorption correction	Multi-scan (*CrysAlis PRO*; Rigaku OD, 2023 ▸)	Multi-scan (*CrysAlis PRO*; Rigaku OD, 2023 ▸)	Multi-scan (*CrysAlis PRO*; Rigaku OD, 2023 ▸)
*T*_min_, *T*_max_	0.434, 1.000	0.687, 1.000	0.631, 1.000
No. of measured, independent and observed [*I* > 2σ(*I*)] reflections	19726, 6526, 5354	11463, 3719, 3369	35740, 6507, 5739
*R*_int_	0.040	0.035	0.038
(sin θ/λ)_max_ (Å^−1^)	0.615	0.608	0.609

Refinement			
*R*[*F*^2^ > 2σ(*F*^2^)], *wR*(*F*^2^), *S*	0.057, 0.170, 1.06	0.030, 0.070, 1.02	0.033, 0.086, 1.05
No. of reflections	6526	3719	6507
No. of parameters	389	232	418
H-atom treatment	H-atom parameters constrained	Only H-atom displacement parameters refined	Only H-atom displacement parameters refined
Δρ_max_, Δρ_min_ (e Å^−3^)	0.67, −0.27	0.28, −0.31	0.50, −0.25

Computer programs: *CrysAlis PRO* (Rigaku OD, 2023 ▸), *SHELXT2018/2* (Sheldrick, 2015*a* ▸), *SHELXL2018/3* (Sheldrick, 2015*b* ▸) and *OLEX2* (Dolomanov *et al.*, 2009 ▸).

## supplementary crystallographic information

### Bis(triphenylsulfonium) tetrachloridomanganate(II) (I) Crystal data

**Table:**

(C_18_H_15_S)_2_[MnCl_4_]	*F*(000) = 1484
*M**_r_* = 723.46	*D*_x_ = 1.370 Mg m^−^^3^
Monoclinic, *P*2_1_/*n*	Cu *K*α radiation, λ = 1.54184 Å
*a* = 9.3550 (4) Å	Cell parameters from 10821 reflections
*b* = 17.7628 (8) Å	θ = 3.2–69.7°
*c* = 21.3952 (9) Å	µ = 7.16 mm^−^^1^
β = 99.465 (4)°	*T* = 297 K
*V* = 3506.9 (3) Å^3^	Block, clear colourless
*Z* = 4	0.22 × 0.14 × 0.06 mm

### Bis(triphenylsulfonium) tetrachloridomanganate(II) (I) Data collection

**Table:**

XtaLAB Synergy, Single source at home/near, HyPix3000 diffractometer	6526 independent reflections
Radiation source: micro-focus sealed X-ray tube, PhotonJet (Cu) X-ray Source	5354 reflections with *I* > 2σ(*I*)
Mirror monochromator	*R*_int_ = 0.040
Detector resolution: 10.0000 pixels mm^-1^	θ_max_ = 71.4°, θ_min_ = 3.3°
ω scans	*h* = −11→11
Absorption correction: multi-scan (CrysAlisPro; Rigaku OD, 2023)	*k* = −15→21
*T*_min_ = 0.434, *T*_max_ = 1.000	*l* = −26→25
19726 measured reflections	

### Bis(triphenylsulfonium) tetrachloridomanganate(II) (I) Refinement

**Table:**

Refinement on *F*^2^	Hydrogen site location: inferred from neighbouring sites
Least-squares matrix: full	H-atom parameters constrained
*R*[*F*^2^ > 2σ(*F*^2^)] = 0.057	*w* = 1/[σ^2^(*F*_o_^2^) + (0.057*P*)^2^ + 8.8484*P*] where *P* = (*F*_o_^2^ + 2*F*_c_^2^)/3
*wR*(*F*^2^) = 0.170	(Δ/σ)_max_ = 0.001
*S* = 1.06	Δρ_max_ = 0.67 e Å^−^^3^
6526 reflections	Δρ_min_ = −0.27 e Å^−^^3^
389 parameters	Extinction correction: SHELXL-2018/3 (Sheldrick 2015b), Fc^*^=kFc[1+0.001xFc^2^λ^3^/sin(2θ)]^-1/4^
0 restraints	Extinction coefficient: 0.00044 (9)
Primary atom site location: dual	

### Bis(triphenylsulfonium) tetrachloridomanganate(II) (I) Special details

**Table:**

Geometry. All esds (except the esd in the dihedral angle between two l.s. planes) are estimated using the full covariance matrix. The cell esds are taken into account individually in the estimation of esds in distances, angles and torsion angles; correlations between esds in cell parameters are only used when they are defined by crystal symmetry. An approximate (isotropic) treatment of cell esds is used for estimating esds involving l.s. planes.

### Bis(triphenylsulfonium) tetrachloridomanganate(II) (I) Fractional atomic coordinates and isotropic or equivalent isotropic displacement parameters (Å^2^)

**Table:**

	*x*	*y*	*z*	*U*_iso_*/*U*_eq_	
C1	0.3691 (5)	0.3639 (3)	0.4982 (2)	0.0491 (11)	
Cl1	0.58908 (16)	0.10720 (9)	0.61299 (7)	0.0751 (4)	
Mn1	0.45825 (8)	0.19317 (4)	0.66447 (3)	0.0447 (2)	
S1	0.45857 (12)	0.27483 (6)	0.50262 (5)	0.0443 (3)	
C2	0.2801 (6)	0.3751 (3)	0.5426 (3)	0.0629 (13)	
H2	0.269284	0.337695	0.571899	0.075*	
Cl2	0.56721 (16)	0.31339 (7)	0.66503 (6)	0.0666 (4)	
S2	0.74651 (13)	−0.01144 (7)	0.31301 (5)	0.0490 (3)	
C3	0.2071 (7)	0.4428 (4)	0.5429 (3)	0.0827 (18)	
H3	0.144658	0.450821	0.571895	0.099*	
Cl3	0.45788 (18)	0.15745 (8)	0.77075 (6)	0.0720 (4)	
C4	0.2271 (8)	0.4987 (3)	0.5000 (3)	0.0830 (19)	
H4	0.178356	0.544252	0.500449	0.100*	
Cl4	0.22288 (13)	0.19429 (8)	0.60244 (6)	0.0624 (3)	
C5	0.3173 (9)	0.4873 (4)	0.4576 (3)	0.091 (2)	
H5	0.330627	0.525401	0.429279	0.110*	
C6	0.3901 (7)	0.4195 (3)	0.4557 (3)	0.0730 (16)	
H6	0.451783	0.411762	0.426356	0.088*	
C7	0.3708 (5)	0.2212 (3)	0.4363 (2)	0.0459 (10)	
C8	0.2774 (6)	0.2513 (3)	0.3862 (2)	0.0606 (13)	
H8	0.248756	0.301460	0.386321	0.073*	
C9	0.2275 (7)	0.2047 (4)	0.3356 (3)	0.0724 (16)	
H9	0.165640	0.223909	0.300750	0.087*	
C10	0.2678 (7)	0.1314 (4)	0.3361 (3)	0.0721 (16)	
H10	0.234321	0.101232	0.301265	0.087*	
C11	0.3570 (7)	0.1008 (3)	0.3872 (3)	0.0723 (16)	
H11	0.381939	0.050105	0.387213	0.087*	
C12	0.4099 (6)	0.1461 (3)	0.4389 (3)	0.0605 (13)	
H12	0.469823	0.126436	0.474076	0.073*	
C13	0.6326 (5)	0.2907 (3)	0.4809 (2)	0.0496 (11)	
C14	0.6493 (7)	0.3010 (4)	0.4189 (3)	0.0763 (17)	
H14	0.569577	0.302174	0.386573	0.092*	
C15	0.7902 (8)	0.3095 (4)	0.4059 (3)	0.090 (2)	
H15	0.805264	0.317040	0.364413	0.108*	
C16	0.9061 (7)	0.3068 (4)	0.4543 (4)	0.088 (2)	
H16	0.999402	0.312539	0.445261	0.106*	
C17	0.8869 (6)	0.2960 (3)	0.5148 (4)	0.0741 (17)	
H17	0.966709	0.293893	0.547051	0.089*	
C18	0.7496 (5)	0.2880 (3)	0.5290 (3)	0.0573 (12)	
H18	0.736014	0.280793	0.570647	0.069*	
C19	0.8985 (5)	−0.0307 (3)	0.2744 (2)	0.0522 (11)	
C20	0.9769 (6)	−0.0944 (4)	0.2943 (3)	0.0717 (16)	
H20	0.949863	−0.124624	0.325873	0.086*	
C21	1.0954 (7)	−0.1130 (4)	0.2670 (3)	0.0785 (17)	
H21	1.147716	−0.156419	0.279582	0.094*	
C22	1.1364 (7)	−0.0675 (4)	0.2214 (3)	0.0791 (18)	
H22	1.216963	−0.079843	0.203135	0.095*	
C23	1.0599 (9)	−0.0048 (4)	0.2031 (3)	0.096 (2)	
H23	1.089420	0.026129	0.172535	0.115*	
C24	0.9367 (8)	0.0148 (3)	0.2289 (3)	0.0799 (19)	
H24	0.883029	0.057526	0.215287	0.096*	
C25	0.8234 (5)	0.0300 (3)	0.3869 (2)	0.0482 (10)	
C26	0.7434 (6)	0.0199 (3)	0.4347 (3)	0.0607 (13)	
H26	0.659308	−0.008959	0.428363	0.073*	
C27	0.7915 (7)	0.0540 (4)	0.4923 (3)	0.0746 (17)	
H27	0.738411	0.049102	0.525235	0.089*	
C28	0.9181 (7)	0.0951 (3)	0.5013 (3)	0.0721 (16)	
H28	0.949294	0.118201	0.540134	0.087*	
C29	0.9975 (7)	0.1024 (3)	0.4542 (3)	0.0701 (15)	
H29	1.083797	0.129394	0.461110	0.084*	
C30	0.9502 (6)	0.0694 (3)	0.3953 (3)	0.0622 (13)	
H30	1.003870	0.074123	0.362615	0.075*	
C31	0.6536 (5)	0.0650 (3)	0.2697 (2)	0.0532 (11)	
C32	0.5290 (7)	0.0468 (4)	0.2296 (3)	0.0776 (18)	
H32	0.498171	−0.002946	0.225291	0.093*	
C33	0.4488 (8)	0.1037 (4)	0.1954 (4)	0.094 (2)	
H33	0.362919	0.092471	0.168451	0.113*	
C34	0.4978 (8)	0.1765 (4)	0.2017 (3)	0.0822 (19)	
H34	0.445876	0.214552	0.178165	0.099*	
C35	0.6210 (9)	0.1937 (4)	0.2419 (3)	0.088 (2)	
H35	0.652164	0.243412	0.245823	0.106*	
C36	0.7011 (7)	0.1383 (3)	0.2770 (3)	0.0763 (17)	
H36	0.785015	0.150257	0.304957	0.092*	

### Bis(triphenylsulfonium) tetrachloridomanganate(II) (I) Atomic displacement parameters (Å^2^)

**Table:**

	*U*^11^	*U*^22^	*U*^33^	*U*^12^	*U*^13^	*U*^23^
C1	0.049 (3)	0.047 (2)	0.049 (2)	0.001 (2)	0.002 (2)	−0.003 (2)
Cl1	0.0742 (9)	0.0779 (9)	0.0744 (9)	0.0204 (7)	0.0161 (7)	−0.0235 (7)
Mn1	0.0469 (4)	0.0461 (4)	0.0411 (4)	−0.0019 (3)	0.0072 (3)	−0.0074 (3)
S1	0.0447 (6)	0.0470 (6)	0.0407 (5)	0.0025 (5)	0.0056 (4)	0.0009 (4)
C2	0.061 (3)	0.055 (3)	0.075 (3)	0.007 (3)	0.016 (3)	0.002 (3)
Cl2	0.0798 (9)	0.0591 (7)	0.0582 (7)	−0.0221 (7)	0.0031 (6)	−0.0099 (6)
S2	0.0494 (6)	0.0482 (6)	0.0473 (6)	−0.0065 (5)	0.0020 (5)	−0.0016 (5)
C3	0.081 (4)	0.071 (4)	0.100 (5)	0.021 (3)	0.027 (4)	−0.006 (4)
Cl3	0.1020 (11)	0.0715 (8)	0.0456 (6)	−0.0151 (8)	0.0212 (7)	−0.0051 (6)
C4	0.093 (5)	0.051 (3)	0.103 (5)	0.014 (3)	0.009 (4)	−0.001 (3)
Cl4	0.0473 (6)	0.0660 (8)	0.0703 (8)	−0.0069 (6)	−0.0012 (6)	0.0034 (6)
C5	0.130 (6)	0.057 (4)	0.085 (5)	0.014 (4)	0.011 (4)	0.013 (3)
C6	0.095 (5)	0.061 (3)	0.066 (3)	0.011 (3)	0.019 (3)	0.009 (3)
C7	0.046 (2)	0.047 (2)	0.043 (2)	−0.001 (2)	0.0038 (19)	−0.0022 (19)
C8	0.063 (3)	0.061 (3)	0.055 (3)	0.006 (3)	0.000 (2)	0.006 (2)
C9	0.073 (4)	0.086 (4)	0.051 (3)	0.004 (3)	−0.009 (3)	0.000 (3)
C10	0.069 (4)	0.086 (4)	0.060 (3)	−0.012 (3)	0.008 (3)	−0.025 (3)
C11	0.073 (4)	0.058 (3)	0.084 (4)	0.004 (3)	0.008 (3)	−0.018 (3)
C12	0.057 (3)	0.054 (3)	0.065 (3)	0.013 (2)	−0.006 (2)	−0.002 (2)
C13	0.051 (3)	0.049 (2)	0.052 (3)	−0.004 (2)	0.014 (2)	−0.008 (2)
C14	0.071 (4)	0.102 (5)	0.059 (3)	−0.017 (3)	0.022 (3)	−0.009 (3)
C15	0.083 (5)	0.118 (6)	0.081 (4)	−0.019 (4)	0.047 (4)	−0.020 (4)
C16	0.065 (4)	0.074 (4)	0.137 (7)	−0.009 (3)	0.050 (5)	−0.022 (4)
C17	0.048 (3)	0.068 (4)	0.105 (5)	0.000 (3)	0.010 (3)	0.006 (3)
C18	0.049 (3)	0.054 (3)	0.069 (3)	0.004 (2)	0.011 (2)	0.005 (2)
C19	0.056 (3)	0.050 (3)	0.049 (3)	−0.006 (2)	0.002 (2)	−0.007 (2)
C20	0.064 (3)	0.081 (4)	0.073 (4)	0.009 (3)	0.020 (3)	0.018 (3)
C21	0.058 (3)	0.090 (4)	0.087 (4)	0.009 (3)	0.012 (3)	0.007 (4)
C22	0.076 (4)	0.079 (4)	0.090 (4)	−0.014 (3)	0.035 (3)	−0.027 (4)
C23	0.139 (7)	0.070 (4)	0.095 (5)	0.001 (4)	0.069 (5)	0.006 (4)
C24	0.113 (5)	0.062 (3)	0.076 (4)	0.008 (3)	0.047 (4)	0.009 (3)
C25	0.048 (3)	0.050 (2)	0.045 (2)	0.001 (2)	0.0027 (19)	−0.005 (2)
C26	0.052 (3)	0.071 (3)	0.061 (3)	0.006 (3)	0.012 (2)	−0.002 (3)
C27	0.082 (4)	0.091 (4)	0.053 (3)	0.016 (4)	0.019 (3)	−0.011 (3)
C28	0.087 (4)	0.067 (3)	0.055 (3)	0.013 (3)	−0.007 (3)	−0.019 (3)
C29	0.068 (4)	0.064 (3)	0.072 (4)	−0.012 (3)	−0.009 (3)	−0.011 (3)
C30	0.059 (3)	0.072 (3)	0.056 (3)	−0.013 (3)	0.008 (2)	−0.006 (3)
C31	0.049 (3)	0.055 (3)	0.052 (3)	−0.003 (2)	−0.001 (2)	0.001 (2)
C32	0.071 (4)	0.066 (4)	0.083 (4)	−0.007 (3)	−0.023 (3)	0.003 (3)
C33	0.079 (4)	0.088 (5)	0.098 (5)	0.001 (4)	−0.034 (4)	0.006 (4)
C34	0.086 (5)	0.075 (4)	0.078 (4)	0.015 (4)	−0.008 (4)	0.016 (3)
C35	0.109 (6)	0.056 (3)	0.092 (5)	0.005 (4)	−0.006 (4)	0.014 (3)
C36	0.076 (4)	0.059 (3)	0.083 (4)	−0.003 (3)	−0.019 (3)	0.005 (3)

### Bis(triphenylsulfonium) tetrachloridomanganate(II) (I) Geometric parameters (Å, º)

**Table:**

C1—S1	1.785 (5)	C16—H16	0.9300
C1—C2	1.376 (7)	C16—C17	1.351 (10)
C1—C6	1.378 (7)	C17—H17	0.9300
Cl1—Mn1	2.3421 (14)	C17—C18	1.374 (8)
Mn1—Cl2	2.3655 (14)	C18—H18	0.9300
Mn1—Cl3	2.3613 (14)	C19—C20	1.377 (8)
Mn1—Cl4	2.3768 (14)	C19—C24	1.358 (7)
S1—C7	1.793 (5)	C20—H20	0.9300
S1—C13	1.788 (5)	C20—C21	1.375 (8)
C2—H2	0.9300	C21—H21	0.9300
C2—C3	1.385 (8)	C21—C22	1.370 (9)
S2—C19	1.791 (5)	C22—H22	0.9300
S2—C25	1.785 (5)	C22—C23	1.346 (10)
S2—C31	1.787 (5)	C23—H23	0.9300
C3—H3	0.9300	C23—C24	1.401 (9)
C3—C4	1.385 (9)	C24—H24	0.9300
C4—H4	0.9300	C25—C26	1.374 (7)
C4—C5	1.353 (10)	C25—C30	1.363 (7)
C5—H5	0.9300	C26—H26	0.9300
C5—C6	1.387 (9)	C26—C27	1.381 (8)
C6—H6	0.9300	C27—H27	0.9300
C7—C8	1.376 (7)	C27—C28	1.378 (9)
C7—C12	1.381 (7)	C28—H28	0.9300
C8—H8	0.9300	C28—C29	1.352 (9)
C8—C9	1.381 (8)	C29—H29	0.9300
C9—H9	0.9300	C29—C30	1.393 (7)
C9—C10	1.356 (9)	C30—H30	0.9300
C10—H10	0.9300	C31—C32	1.367 (7)
C10—C11	1.373 (8)	C31—C36	1.375 (8)
C11—H11	0.9300	C32—H32	0.9300
C11—C12	1.391 (7)	C32—C33	1.393 (9)
C12—H12	0.9300	C33—H33	0.9300
C13—C14	1.373 (7)	C33—C34	1.371 (9)
C13—C18	1.374 (7)	C34—H34	0.9300
C14—H14	0.9300	C34—C35	1.354 (9)
C14—C15	1.400 (9)	C35—H35	0.9300
C15—H15	0.9300	C35—C36	1.382 (8)
C15—C16	1.371 (10)	C36—H36	0.9300
			
C2—C1—S1	115.0 (4)	C16—C17—H17	119.9
C2—C1—C6	121.3 (5)	C16—C17—C18	120.2 (6)
C6—C1—S1	123.6 (4)	C18—C17—H17	119.9
Cl1—Mn1—Cl2	109.44 (6)	C13—C18—H18	120.3
Cl1—Mn1—Cl3	111.18 (6)	C17—C18—C13	119.3 (5)
Cl1—Mn1—Cl4	104.52 (6)	C17—C18—H18	120.3
Cl2—Mn1—Cl4	110.74 (6)	C20—C19—S2	115.9 (4)
Cl3—Mn1—Cl2	107.85 (5)	C24—C19—S2	122.7 (4)
Cl3—Mn1—Cl4	113.08 (6)	C24—C19—C20	121.4 (5)
C1—S1—C7	106.2 (2)	C19—C20—H20	120.3
C1—S1—C13	106.6 (2)	C21—C20—C19	119.4 (6)
C13—S1—C7	101.3 (2)	C21—C20—H20	120.3
C1—C2—H2	120.5	C20—C21—H21	120.0
C1—C2—C3	118.9 (5)	C22—C21—C20	120.1 (6)
C3—C2—H2	120.5	C22—C21—H21	120.0
C25—S2—C19	104.7 (2)	C21—C22—H22	120.1
C25—S2—C31	103.7 (2)	C23—C22—C21	119.8 (6)
C31—S2—C19	105.2 (2)	C23—C22—H22	120.1
C2—C3—H3	120.0	C22—C23—H23	119.2
C4—C3—C2	119.9 (6)	C22—C23—C24	121.6 (6)
C4—C3—H3	120.0	C24—C23—H23	119.2
C3—C4—H4	119.8	C19—C24—C23	117.7 (6)
C5—C4—C3	120.3 (6)	C19—C24—H24	121.2
C5—C4—H4	119.8	C23—C24—H24	121.2
C4—C5—H5	119.6	C26—C25—S2	114.5 (4)
C4—C5—C6	120.8 (6)	C30—C25—S2	122.9 (4)
C6—C5—H5	119.6	C30—C25—C26	122.5 (5)
C1—C6—C5	118.6 (6)	C25—C26—H26	121.0
C1—C6—H6	120.7	C25—C26—C27	118.0 (5)
C5—C6—H6	120.7	C27—C26—H26	121.0
C8—C7—S1	124.0 (4)	C26—C27—H27	119.9
C8—C7—C12	122.5 (5)	C28—C27—C26	120.2 (6)
C12—C7—S1	113.4 (4)	C28—C27—H27	119.9
C7—C8—H8	121.1	C27—C28—H28	119.6
C7—C8—C9	117.8 (5)	C29—C28—C27	120.8 (5)
C9—C8—H8	121.1	C29—C28—H28	119.6
C8—C9—H9	119.6	C28—C29—H29	119.9
C10—C9—C8	120.8 (5)	C28—C29—C30	120.1 (5)
C10—C9—H9	119.6	C30—C29—H29	119.9
C9—C10—H10	119.4	C25—C30—C29	118.4 (5)
C9—C10—C11	121.3 (5)	C25—C30—H30	120.8
C11—C10—H10	119.4	C29—C30—H30	120.8
C10—C11—H11	120.2	C32—C31—S2	115.9 (4)
C10—C11—C12	119.6 (5)	C32—C31—C36	121.4 (5)
C12—C11—H11	120.2	C36—C31—S2	122.7 (4)
C7—C12—C11	118.0 (5)	C31—C32—H32	120.4
C7—C12—H12	121.0	C31—C32—C33	119.2 (6)
C11—C12—H12	121.0	C33—C32—H32	120.4
C14—C13—S1	121.7 (4)	C32—C33—H33	120.3
C14—C13—C18	121.7 (5)	C34—C33—C32	119.3 (6)
C18—C13—S1	116.5 (4)	C34—C33—H33	120.3
C13—C14—H14	121.1	C33—C34—H34	119.7
C13—C14—C15	117.8 (6)	C35—C34—C33	120.7 (6)
C15—C14—H14	121.1	C35—C34—H34	119.7
C14—C15—H15	120.0	C34—C35—H35	119.5
C16—C15—C14	119.9 (6)	C34—C35—C36	120.9 (6)
C16—C15—H15	120.0	C36—C35—H35	119.5
C15—C16—H16	119.5	C31—C36—C35	118.4 (6)
C17—C16—C15	121.1 (6)	C31—C36—H36	120.8
C17—C16—H16	119.5	C35—C36—H36	120.8
			
C1—S1—C7—C8	13.1 (5)	C13—C14—C15—C16	0.6 (11)
C1—S1—C7—C12	−169.2 (4)	C14—C13—C18—C17	0.2 (8)
C1—S1—C13—C14	−76.4 (5)	C14—C15—C16—C17	0.0 (11)
C1—S1—C13—C18	107.1 (4)	C15—C16—C17—C18	−0.5 (10)
C1—C2—C3—C4	−1.6 (10)	C16—C17—C18—C13	0.4 (9)
S1—C1—C2—C3	179.6 (5)	C18—C13—C14—C15	−0.7 (9)
S1—C1—C6—C5	−178.4 (5)	C19—S2—C25—C26	−153.0 (4)
S1—C7—C8—C9	174.4 (4)	C19—S2—C25—C30	27.8 (5)
S1—C7—C12—C11	−174.9 (4)	C19—S2—C31—C32	104.0 (5)
S1—C13—C14—C15	−177.1 (5)	C19—S2—C31—C36	−77.6 (6)
S1—C13—C18—C17	176.8 (4)	C19—C20—C21—C22	1.3 (10)
C2—C1—S1—C7	107.0 (4)	C20—C19—C24—C23	−0.6 (10)
C2—C1—S1—C13	−145.5 (4)	C20—C21—C22—C23	−0.4 (11)
C2—C1—C6—C5	−1.2 (9)	C21—C22—C23—C24	−1.0 (12)
C2—C3—C4—C5	0.3 (11)	C22—C23—C24—C19	1.5 (11)
S2—C19—C20—C21	−179.8 (5)	C24—C19—C20—C21	−0.8 (9)
S2—C19—C24—C23	178.4 (5)	C25—S2—C19—C20	79.3 (5)
S2—C25—C26—C27	−176.4 (4)	C25—S2—C19—C24	−99.7 (5)
S2—C25—C30—C29	177.2 (4)	C25—S2—C31—C32	−146.4 (5)
S2—C31—C32—C33	178.5 (6)	C25—S2—C31—C36	32.0 (6)
S2—C31—C36—C35	−179.4 (5)	C25—C26—C27—C28	−1.5 (9)
C3—C4—C5—C6	0.6 (12)	C26—C25—C30—C29	−2.0 (8)
C4—C5—C6—C1	−0.2 (11)	C26—C27—C28—C29	−0.5 (9)
C6—C1—S1—C7	−75.6 (5)	C27—C28—C29—C30	1.4 (9)
C6—C1—S1—C13	31.9 (5)	C28—C29—C30—C25	−0.1 (9)
C6—C1—C2—C3	2.1 (9)	C30—C25—C26—C27	2.8 (8)
C7—S1—C13—C14	34.5 (5)	C31—S2—C19—C20	−171.8 (4)
C7—S1—C13—C18	−142.0 (4)	C31—S2—C19—C24	9.1 (5)
C7—C8—C9—C10	1.1 (9)	C31—S2—C25—C26	97.1 (4)
C8—C7—C12—C11	2.9 (8)	C31—S2—C25—C30	−82.2 (5)
C8—C9—C10—C11	1.1 (10)	C31—C32—C33—C34	1.1 (12)
C9—C10—C11—C12	−1.4 (10)	C32—C31—C36—C35	−1.1 (10)
C10—C11—C12—C7	−0.6 (9)	C32—C33—C34—C35	−1.5 (12)
C12—C7—C8—C9	−3.2 (8)	C33—C34—C35—C36	0.6 (12)
C13—S1—C7—C8	−98.1 (5)	C34—C35—C36—C31	0.8 (12)
C13—S1—C7—C12	79.6 (4)	C36—C31—C32—C33	0.2 (10)

### Bis(triphenylsulfonium) tetrachloridomanganate(II) (I) Hydrogen-bond geometry (Å, º)

**Table:**

*D*—H···*A*	*D*—H	H···*A*	*D*···*A*	*D*—H···*A*
C2—H2···Cl4	0.93	2.68	3.530 (6)	152
C6—H6···C14	0.93	2.72	3.401 (9)	131
C8—H8···C6	0.93	2.78	3.425 (8)	128
C9—H9···Cl3^i^	0.93	2.87	3.624 (6)	139
C14—H14···C8	0.93	2.88	3.546 (8)	130
C15—H15···Cl3^ii^	0.93	2.68	3.557 (6)	157
C18—H18···Cl2	0.93	2.82	3.636 (6)	147
C20—H20···Cl4^iii^	0.93	2.70	3.589 (6)	159
C23—H23···Cl2^ii^	0.93	2.86	3.500 (7)	127
C24—H24···C36	0.93	2.73	3.389 (9)	129
C27—H27···Cl1	0.93	2.72	3.571 (6)	152
C30—H30···C10^iv^	0.93	2.81	3.589 (8)	142
C30—H30···C24	0.93	3.02	3.674 (8)	129
C32—H32···Cl3^iii^	0.93	2.77	3.631 (7)	154
C36—H36···C30	0.93	2.68	3.376 (8)	132

Symmetry codes: (i) *x*−1/2, −*y*+1/2, *z*−1/2; (ii) *x*+1/2, −*y*+1/2, *z*−1/2; (iii) −*x*+1, −*y*, −*z*+1; (iv) *x*+1, *y*, *z*.

### Triphenylsulfonium tetrachloridoferrate(III) (II) Crystal data

**Table:**

(C_18_H_15_S)[FeCl_4_]	*F*(000) = 932
*M**_r_* = 461.01	*D*_x_ = 1.532 Mg m^−^^3^
Monoclinic, *P*2_1_/*c*	Cu *K*α radiation, λ = 1.54184 Å
*a* = 8.3336 (1) Å	Cell parameters from 6438 reflections
*b* = 15.2143 (2) Å	θ = 4.0–69.5°
*c* = 16.0128 (2) Å	µ = 11.92 mm^−^^1^
β = 100.175 (1)°	*T* = 100 K
*V* = 1998.33 (4) Å^3^	Irregular, clear light yellow
*Z* = 4	0.18 × 0.15 × 0.10 mm

### Triphenylsulfonium tetrachloridoferrate(III) (II) Data collection

**Table:**

XtaLAB Synergy, Single source at home/near, HyPix3000 diffractometer	3369 reflections with *I* > 2σ(*I*)
Detector resolution: 10.0000 pixels mm^-1^	*R*_int_ = 0.035
ω scans	θ_max_ = 69.7°, θ_min_ = 4.0°
Absorption correction: multi-scan (CrysAlisPro; Rigaku OD, 2023)	*h* = −9→10
*T*_min_ = 0.687, *T*_max_ = 1.000	*k* = −18→18
11463 measured reflections	*l* = −19→14
3719 independent reflections	

### Triphenylsulfonium tetrachloridoferrate(III) (II) Refinement

**Table:**

Refinement on *F*^2^	0 restraints
Least-squares matrix: full	Hydrogen site location: inferred from neighbouring sites
*R*[*F*^2^ > 2σ(*F*^2^)] = 0.030	Only H-atom displacement parameters refined
*wR*(*F*^2^) = 0.070	*w* = 1/[σ^2^(*F*_o_^2^) + (0.0293*P*)^2^ + 1.1416*P*] where *P* = (*F*_o_^2^ + 2*F*_c_^2^)/3
*S* = 1.02	(Δ/σ)_max_ = 0.001
3719 reflections	Δρ_max_ = 0.28 e Å^−^^3^
232 parameters	Δρ_min_ = −0.31 e Å^−^^3^

### Triphenylsulfonium tetrachloridoferrate(III) (II) Special details

**Table:**

Geometry. All esds (except the esd in the dihedral angle between two l.s. planes) are estimated using the full covariance matrix. The cell esds are taken into account individually in the estimation of esds in distances, angles and torsion angles; correlations between esds in cell parameters are only used when they are defined by crystal symmetry. An approximate (isotropic) treatment of cell esds is used for estimating esds involving l.s. planes.

### Triphenylsulfonium tetrachloridoferrate(III) (II) Fractional atomic coordinates and isotropic or equivalent isotropic displacement parameters (Å^2^)

**Table:**

	*x*	*y*	*z*	*U*_iso_*/*U*_eq_	
C1	0.6471 (3)	0.54745 (14)	0.84124 (14)	0.0182 (4)	
Cl1	0.90172 (7)	0.74214 (4)	0.50286 (4)	0.02956 (14)	
Fe1	0.76602 (4)	0.70241 (2)	0.60214 (2)	0.01644 (10)	
S1	0.53518 (6)	0.61905 (3)	0.76368 (3)	0.01540 (12)	
C2	0.6486 (3)	0.55868 (15)	0.92735 (15)	0.0217 (5)	
H2	0.587958	0.604572	0.947362	0.037 (8)*	
Cl2	0.55921 (6)	0.79242 (3)	0.60243 (3)	0.02146 (12)	
C3	0.7410 (3)	0.50105 (17)	0.98344 (15)	0.0278 (5)	
H3	0.745238	0.508025	1.042743	0.033 (8)*	
Cl3	0.92474 (7)	0.70798 (4)	0.72729 (4)	0.02715 (14)	
C4	0.8271 (3)	0.43354 (17)	0.95390 (16)	0.0287 (5)	
H4	0.888907	0.394103	0.992960	0.038 (8)*	
Cl4	0.67743 (6)	0.56700 (3)	0.57817 (3)	0.02001 (12)	
C5	0.8237 (3)	0.42315 (17)	0.86757 (17)	0.0304 (6)	
H5	0.882357	0.376377	0.847596	0.041 (8)*	
C6	0.7345 (3)	0.48120 (16)	0.81018 (15)	0.0245 (5)	
H6	0.733447	0.475554	0.750999	0.020 (6)*	
C7	0.3590 (3)	0.55742 (14)	0.71933 (14)	0.0162 (4)	
C8	0.3092 (3)	0.48398 (14)	0.75993 (14)	0.0199 (5)	
H8	0.369290	0.464168	0.812555	0.017 (6)*	
C9	0.1692 (3)	0.44060 (15)	0.72114 (16)	0.0240 (5)	
H9	0.132539	0.390286	0.747442	0.030 (7)*	
C10	0.0826 (3)	0.47005 (16)	0.64445 (16)	0.0250 (5)	
H10	−0.013113	0.439875	0.618533	0.034 (8)*	
C11	0.1349 (3)	0.54342 (16)	0.60522 (15)	0.0239 (5)	
H11	0.074539	0.563408	0.552728	0.025 (7)*	
C12	0.2747 (3)	0.58757 (15)	0.64232 (13)	0.0190 (4)	
H12	0.311982	0.637418	0.615561	0.019 (6)*	
C13	0.4577 (3)	0.70344 (13)	0.82262 (13)	0.0161 (4)	
C14	0.3122 (3)	0.69458 (15)	0.85209 (14)	0.0205 (5)	
H14	0.248686	0.642609	0.841456	0.029 (7)*	
C15	0.2617 (3)	0.76364 (16)	0.89756 (15)	0.0234 (5)	
H15	0.162076	0.759343	0.918337	0.037 (8)*	
C16	0.3555 (3)	0.83870 (15)	0.91285 (14)	0.0218 (5)	
H16	0.320684	0.885116	0.945094	0.023 (7)*	
C17	0.5004 (3)	0.84723 (15)	0.88162 (13)	0.0209 (5)	
H17	0.563259	0.899482	0.891659	0.033 (7)*	
C18	0.5523 (3)	0.77865 (15)	0.83563 (13)	0.0198 (4)	
H18	0.650592	0.783259	0.813601	0.026 (7)*	

### Triphenylsulfonium tetrachloridoferrate(III) (II) Atomic displacement parameters (Å^2^)

**Table:**

	*U*^11^	*U*^22^	*U*^33^	*U*^12^	*U*^13^	*U*^23^
C1	0.0162 (10)	0.0156 (11)	0.0226 (11)	0.0004 (8)	0.0031 (8)	0.0004 (9)
Cl1	0.0306 (3)	0.0268 (3)	0.0357 (3)	−0.0070 (2)	0.0181 (2)	0.0005 (2)
Fe1	0.01789 (18)	0.01243 (17)	0.01961 (18)	−0.00158 (13)	0.00494 (13)	−0.00184 (13)
S1	0.0174 (2)	0.0129 (2)	0.0163 (2)	0.00025 (18)	0.00403 (19)	−0.00057 (19)
C2	0.0251 (12)	0.0183 (11)	0.0218 (11)	0.0051 (9)	0.0040 (9)	0.0003 (9)
Cl2	0.0244 (3)	0.0169 (3)	0.0231 (3)	0.0034 (2)	0.0045 (2)	−0.0002 (2)
C3	0.0318 (13)	0.0275 (13)	0.0228 (12)	0.0044 (11)	0.0013 (10)	0.0038 (10)
Cl3	0.0243 (3)	0.0259 (3)	0.0281 (3)	0.0035 (2)	−0.0040 (2)	−0.0070 (2)
C4	0.0248 (12)	0.0277 (13)	0.0326 (13)	0.0059 (10)	0.0022 (10)	0.0095 (10)
Cl4	0.0259 (3)	0.0122 (2)	0.0224 (3)	−0.00238 (19)	0.0056 (2)	−0.00146 (19)
C5	0.0279 (13)	0.0262 (13)	0.0384 (14)	0.0116 (11)	0.0089 (11)	0.0027 (11)
C6	0.0271 (12)	0.0238 (12)	0.0238 (11)	0.0064 (10)	0.0074 (9)	−0.0018 (10)
C7	0.0160 (10)	0.0137 (10)	0.0194 (10)	−0.0003 (8)	0.0043 (8)	−0.0051 (8)
C8	0.0226 (11)	0.0153 (11)	0.0228 (11)	0.0017 (9)	0.0067 (9)	0.0020 (9)
C9	0.0242 (12)	0.0152 (11)	0.0345 (13)	−0.0024 (9)	0.0100 (10)	−0.0030 (9)
C10	0.0192 (11)	0.0203 (12)	0.0353 (13)	−0.0022 (9)	0.0042 (10)	−0.0094 (10)
C11	0.0235 (12)	0.0248 (12)	0.0218 (11)	0.0027 (10)	0.0000 (9)	−0.0043 (10)
C12	0.0242 (11)	0.0150 (10)	0.0183 (10)	0.0017 (9)	0.0050 (9)	−0.0005 (8)
C13	0.0207 (11)	0.0119 (10)	0.0154 (10)	0.0044 (8)	0.0024 (8)	0.0016 (8)
C14	0.0185 (11)	0.0163 (11)	0.0268 (11)	−0.0016 (9)	0.0042 (9)	−0.0026 (9)
C15	0.0218 (12)	0.0223 (12)	0.0274 (12)	0.0048 (9)	0.0078 (10)	0.0005 (10)
C16	0.0313 (13)	0.0153 (11)	0.0191 (11)	0.0060 (9)	0.0049 (9)	0.0001 (9)
C17	0.0305 (12)	0.0136 (10)	0.0182 (10)	−0.0034 (9)	0.0033 (9)	−0.0002 (9)
C18	0.0236 (11)	0.0186 (11)	0.0178 (10)	−0.0009 (9)	0.0050 (9)	0.0020 (9)

### Triphenylsulfonium tetrachloridoferrate(III) (II) Geometric parameters (Å, º)

**Table:**

C1—S1	1.786 (2)	C8—H8	0.9500
C1—C2	1.387 (3)	C8—C9	1.388 (3)
C1—C6	1.386 (3)	C9—H9	0.9500
Cl1—Fe1	2.1923 (6)	C9—C10	1.384 (4)
Fe1—Cl2	2.2020 (6)	C10—H10	0.9500
Fe1—Cl3	2.1993 (6)	C10—C11	1.388 (3)
Fe1—Cl4	2.1992 (6)	C11—H11	0.9500
S1—C7	1.781 (2)	C11—C12	1.384 (3)
S1—C13	1.781 (2)	C12—H12	0.9500
C2—H2	0.9500	C13—C14	1.383 (3)
C2—C3	1.387 (3)	C13—C18	1.384 (3)
C3—H3	0.9500	C14—H14	0.9500
C3—C4	1.383 (4)	C14—C15	1.385 (3)
C4—H4	0.9500	C15—H15	0.9500
C4—C5	1.387 (4)	C15—C16	1.381 (3)
C5—H5	0.9500	C16—H16	0.9500
C5—C6	1.392 (3)	C16—C17	1.392 (3)
C6—H6	0.9500	C17—H17	0.9500
C7—C8	1.392 (3)	C17—C18	1.389 (3)
C7—C12	1.385 (3)	C18—H18	0.9500
			
C2—C1—S1	121.87 (17)	C9—C8—H8	121.0
C6—C1—S1	115.90 (17)	C8—C9—H9	119.7
C6—C1—C2	122.2 (2)	C10—C9—C8	120.6 (2)
Cl1—Fe1—Cl2	109.53 (3)	C10—C9—H9	119.7
Cl1—Fe1—Cl3	110.23 (3)	C9—C10—H10	119.8
Cl1—Fe1—Cl4	109.39 (2)	C9—C10—C11	120.4 (2)
Cl3—Fe1—Cl2	108.61 (2)	C11—C10—H10	119.8
Cl4—Fe1—Cl2	110.13 (2)	C10—C11—H11	119.9
Cl4—Fe1—Cl3	108.93 (3)	C12—C11—C10	120.2 (2)
C7—S1—C1	104.39 (10)	C12—C11—H11	119.9
C7—S1—C13	103.90 (10)	C7—C12—H12	120.7
C13—S1—C1	105.36 (10)	C11—C12—C7	118.5 (2)
C1—C2—H2	120.9	C11—C12—H12	120.7
C3—C2—C1	118.2 (2)	C14—C13—S1	122.16 (17)
C3—C2—H2	120.9	C14—C13—C18	122.7 (2)
C2—C3—H3	119.7	C18—C13—S1	115.17 (16)
C4—C3—C2	120.7 (2)	C13—C14—H14	120.9
C4—C3—H3	119.7	C13—C14—C15	118.1 (2)
C3—C4—H4	119.8	C15—C14—H14	120.9
C3—C4—C5	120.3 (2)	C14—C15—H15	119.8
C5—C4—H4	119.8	C16—C15—C14	120.4 (2)
C4—C5—H5	120.0	C16—C15—H15	119.8
C4—C5—C6	120.0 (2)	C15—C16—H16	119.6
C6—C5—H5	120.0	C15—C16—C17	120.9 (2)
C1—C6—C5	118.5 (2)	C17—C16—H16	119.6
C1—C6—H6	120.7	C16—C17—H17	120.3
C5—C6—H6	120.7	C18—C17—C16	119.4 (2)
C8—C7—S1	121.93 (17)	C18—C17—H17	120.3
C12—C7—S1	115.68 (16)	C13—C18—C17	118.6 (2)
C12—C7—C8	122.4 (2)	C13—C18—H18	120.7
C7—C8—H8	121.0	C17—C18—H18	120.7
C9—C8—C7	118.0 (2)		
			
C1—S1—C7—C8	17.5 (2)	C6—C1—S1—C13	−173.20 (18)
C1—S1—C7—C12	−162.78 (16)	C6—C1—C2—C3	−0.1 (4)
C1—S1—C13—C14	−86.0 (2)	C7—S1—C13—C14	23.4 (2)
C1—S1—C13—C18	94.82 (18)	C7—S1—C13—C18	−155.70 (17)
C1—C2—C3—C4	−1.0 (4)	C7—C8—C9—C10	0.0 (3)
S1—C1—C2—C3	−178.86 (18)	C8—C7—C12—C11	0.8 (3)
S1—C1—C6—C5	−179.82 (19)	C8—C9—C10—C11	0.1 (3)
S1—C7—C8—C9	179.23 (16)	C9—C10—C11—C12	0.3 (3)
S1—C7—C12—C11	−178.88 (16)	C10—C11—C12—C7	−0.7 (3)
S1—C13—C14—C15	179.86 (17)	C12—C7—C8—C9	−0.4 (3)
S1—C13—C18—C17	−179.56 (16)	C13—S1—C7—C8	−92.67 (18)
C2—C1—S1—C7	−103.5 (2)	C13—S1—C7—C12	87.03 (17)
C2—C1—S1—C13	5.6 (2)	C13—C14—C15—C16	−0.2 (3)
C2—C1—C6—C5	1.4 (4)	C14—C13—C18—C17	1.3 (3)
C2—C3—C4—C5	0.8 (4)	C14—C15—C16—C17	1.3 (4)
C3—C4—C5—C6	0.5 (4)	C15—C16—C17—C18	−1.0 (3)
C4—C5—C6—C1	−1.6 (4)	C16—C17—C18—C13	−0.2 (3)
C6—C1—S1—C7	77.68 (19)	C18—C13—C14—C15	−1.1 (3)

### Triphenylsulfonium tetrachloridoferrate(III) (II) Hydrogen-bond geometry (Å, º)

**Table:**

*D*—H···*A*	*D*—H	H···*A*	*D*···*A*	*D*—H···*A*
C2—H2···C14	0.95	2.87	3.520 (3)	127
C4—H4···Cl1^i^	0.95	2.89	3.676 (3)	141
C14—H14···C8	0.95	2.83	3.526 (3)	131
C15—H15···Cl1^ii^	0.95	2.75	3.693 (2)	171
C16—H16···Cl4^iii^	0.95	2.79	3.489 (2)	131
C18—H18···C9^iii^	0.95	2.93	3.610 (3)	129

Symmetry codes: (i) −*x*+2, *y*−1/2, −*z*+3/2; (ii) *x*−1, −*y*+3/2, *z*+1/2; (iii) −*x*+1, *y*+1/2, −*z*+3/2.

### Bis(triphenylsulfonium) tetrachloridocobaltate(II) (III) Crystal data

**Table:**

(C_18_H_15_S)[CoCl_4_]	*F*(000) = 1492
*M**_r_* = 727.45	*D*_x_ = 1.396 Mg m^−^^3^
Monoclinic, *P*2_1_/*n*	Cu *K*α radiation, λ = 1.54184 Å
*a* = 9.3200 (2) Å	Cell parameters from 19038 reflections
*b* = 17.7236 (3) Å	θ = 3.3–69.5°
*c* = 21.2341 (3) Å	µ = 8.04 mm^−^^1^
β = 99.331 (2)°	*T* = 297 K
*V* = 3461.12 (11) Å^3^	Block, clear bluish colourless
*Z* = 4	0.21 × 0.17 × 0.17 mm

### Bis(triphenylsulfonium) tetrachloridocobaltate(II) (III) Data collection

**Table:**

XtaLAB Synergy, Single source at home/near, HyPix3000 diffractometer	5739 reflections with *I* > 2σ(*I*)
Detector resolution: 10.0000 pixels mm^-1^	*R*_int_ = 0.038
ω scans	θ_max_ = 69.9°, θ_min_ = 3.3°
Absorption correction: multi-scan (CrysAlisPro; Rigaku OD, 2023)	*h* = −11→11
*T*_min_ = 0.631, *T*_max_ = 1.000	*k* = −21→21
35740 measured reflections	*l* = −25→22
6507 independent reflections	

### Bis(triphenylsulfonium) tetrachloridocobaltate(II) (III) Refinement

**Table:**

Refinement on *F*^2^	0 restraints
Least-squares matrix: full	Hydrogen site location: inferred from neighbouring sites
*R*[*F*^2^ > 2σ(*F*^2^)] = 0.033	Only H-atom displacement parameters refined
*wR*(*F*^2^) = 0.086	*w* = 1/[σ^2^(*F*_o_^2^) + (0.0385*P*)^2^ + 1.077*P*] where *P* = (*F*_o_^2^ + 2*F*_c_^2^)/3
*S* = 1.05	(Δ/σ)_max_ = 0.001
6507 reflections	Δρ_max_ = 0.50 e Å^−^^3^
418 parameters	Δρ_min_ = −0.25 e Å^−^^3^

### Bis(triphenylsulfonium) tetrachloridocobaltate(II) (III) Special details

**Table:**

Geometry. All esds (except the esd in the dihedral angle between two l.s. planes) are estimated using the full covariance matrix. The cell esds are taken into account individually in the estimation of esds in distances, angles and torsion angles; correlations between esds in cell parameters are only used when they are defined by crystal symmetry. An approximate (isotropic) treatment of cell esds is used for estimating esds involving l.s. planes.

### Bis(triphenylsulfonium) tetrachloridocobaltate(II) (III) Fractional atomic coordinates and isotropic or equivalent isotropic displacement parameters (Å^2^)

**Table:**

	*x*	*y*	*z*	*U*_iso_*/*U*_eq_	
C1	0.3704 (2)	0.72072 (12)	0.43770 (9)	0.0447 (5)	
Cl1	0.45509 (8)	0.65638 (4)	0.76830 (3)	0.07028 (19)	
Co1	0.45748 (4)	0.69101 (2)	0.66534 (2)	0.04075 (10)	
S1	0.25550 (6)	0.50988 (3)	0.68706 (2)	0.04615 (13)	
C2	0.4083 (3)	0.64573 (14)	0.43910 (12)	0.0602 (6)	
H2	0.467759	0.625361	0.474410	0.063 (7)*	
Cl2	0.58641 (7)	0.60858 (4)	0.61586 (3)	0.07217 (19)	
S2	0.45880 (5)	0.77367 (3)	0.50449 (2)	0.04325 (12)	
C3	0.3562 (3)	0.60119 (16)	0.38689 (14)	0.0725 (7)	
H3	0.382011	0.550529	0.386495	0.091 (10)*	
Cl3	0.23015 (6)	0.69149 (3)	0.60524 (3)	0.06000 (15)	
C4	0.2662 (3)	0.63189 (18)	0.33558 (13)	0.0710 (7)	
H4	0.232715	0.601959	0.300272	0.095 (10)*	
Cl4	0.56082 (7)	0.80750 (3)	0.66708 (3)	0.06352 (17)	
C5	0.2255 (3)	0.70596 (18)	0.33593 (12)	0.0697 (7)	
H5	0.162370	0.725753	0.301464	0.100 (11)*	
C6	0.2777 (3)	0.75147 (15)	0.38731 (11)	0.0577 (6)	
H6	0.250659	0.801955	0.387821	0.063 (8)*	
C7	0.6334 (2)	0.79037 (13)	0.48238 (10)	0.0492 (5)	
C8	0.7496 (3)	0.78767 (14)	0.53115 (12)	0.0563 (6)	
H8	0.735270	0.779938	0.573004	0.080 (9)*	
C9	0.8884 (3)	0.79665 (16)	0.51696 (17)	0.0737 (8)	
H9	0.968284	0.795449	0.549456	0.103 (12)*	
C10	0.9078 (4)	0.80723 (17)	0.45540 (18)	0.0832 (9)	
H10	1.001325	0.813301	0.446185	0.104 (11)*	
C11	0.7921 (4)	0.8091 (2)	0.40710 (16)	0.0905 (11)	
H11	0.807360	0.816331	0.365322	0.110 (12)*	
C12	0.6515 (3)	0.80015 (18)	0.41985 (13)	0.0757 (8)	
H12	0.571990	0.800783	0.387126	0.086 (10)*	
C13	0.3692 (2)	0.86311 (12)	0.50114 (10)	0.0497 (5)	
C14	0.3886 (4)	0.91885 (15)	0.45802 (14)	0.0758 (8)	
H14	0.450957	0.911493	0.428524	0.095 (10)*	
C15	0.3132 (4)	0.98622 (17)	0.45955 (17)	0.0920 (10)	
H15	0.324168	1.024253	0.430537	0.110 (12)*	
C16	0.2228 (4)	0.99694 (17)	0.50349 (17)	0.0863 (9)	
H16	0.171809	1.042010	0.503840	0.086 (9)*	
C17	0.2068 (3)	0.94167 (17)	0.54702 (16)	0.0800 (8)	
H17	0.146579	0.949804	0.577265	0.095 (10)*	
C18	0.2799 (3)	0.87385 (15)	0.54610 (13)	0.0620 (6)	
H18	0.268934	0.836101	0.575368	0.074 (8)*	
C19	0.1031 (2)	0.52798 (13)	0.72650 (10)	0.0476 (5)	
C20	0.0240 (3)	0.59161 (16)	0.70695 (13)	0.0682 (7)	
H20	0.051325	0.622632	0.675604	0.101 (11)*	
C21	−0.0966 (3)	0.60895 (19)	0.73441 (14)	0.0763 (8)	
H21	−0.151952	0.651401	0.721085	0.092 (10)*	
C22	−0.1343 (3)	0.56385 (17)	0.78098 (14)	0.0734 (8)	
H22	−0.214945	0.575845	0.799717	0.085 (9)*	
C23	−0.0554 (4)	0.50196 (18)	0.79999 (16)	0.0918 (11)	
H23	−0.082691	0.471409	0.831637	0.115 (12)*	
C24	0.0658 (4)	0.48294 (16)	0.77326 (13)	0.0745 (8)	
H24	0.120492	0.440386	0.786938	0.099 (11)*	
C25	0.3494 (2)	0.43297 (13)	0.72991 (10)	0.0504 (5)	
C26	0.4759 (3)	0.45081 (16)	0.76959 (13)	0.0709 (7)	
H26	0.508385	0.500462	0.773679	0.065 (8)*	
C27	0.5543 (3)	0.39356 (19)	0.80338 (16)	0.0883 (10)	
H27	0.640516	0.404729	0.830409	0.105 (11)*	
C28	0.5066 (4)	0.32126 (18)	0.79751 (15)	0.0800 (8)	
H28	0.560636	0.283244	0.820434	0.097 (10)*	
C29	0.3803 (4)	0.30376 (17)	0.75836 (15)	0.0800 (9)	
H29	0.348031	0.254049	0.755071	0.100 (11)*	
C30	0.3005 (3)	0.35939 (15)	0.72370 (13)	0.0704 (7)	
H30	0.214861	0.347633	0.696483	0.094 (10)*	
C31	0.1784 (2)	0.46920 (12)	0.61248 (10)	0.0455 (5)	
C32	0.0498 (3)	0.42878 (14)	0.60399 (11)	0.0591 (6)	
H32	−0.003701	0.423520	0.637083	0.076 (8)*	
C33	0.0029 (3)	0.39647 (15)	0.54512 (13)	0.0677 (7)	
H33	−0.083501	0.369258	0.538226	0.091 (10)*	
C34	0.0825 (3)	0.40419 (15)	0.49703 (12)	0.0694 (7)	
H34	0.051221	0.381062	0.457901	0.096 (10)*	
C35	0.2086 (3)	0.44582 (17)	0.50579 (12)	0.0710 (7)	
H35	0.260907	0.451513	0.472343	0.086 (9)*	
C36	0.2580 (3)	0.47934 (15)	0.56420 (11)	0.0565 (6)	
H36	0.342818	0.507926	0.570497	0.058 (7)*	

### Bis(triphenylsulfonium) tetrachloridocobaltate(II) (III) Atomic displacement parameters (Å^2^)

**Table:**

	*U*^11^	*U*^22^	*U*^33^	*U*^12^	*U*^13^	*U*^23^
C1	0.0444 (11)	0.0467 (11)	0.0418 (10)	0.0011 (9)	0.0037 (8)	−0.0001 (9)
Cl1	0.1023 (5)	0.0684 (4)	0.0423 (3)	−0.0148 (4)	0.0181 (3)	−0.0043 (3)
Co1	0.04457 (18)	0.04176 (19)	0.03560 (16)	−0.00141 (14)	0.00558 (13)	−0.00686 (13)
S1	0.0463 (3)	0.0459 (3)	0.0441 (3)	−0.0055 (2)	0.0010 (2)	−0.0014 (2)
C2	0.0606 (14)	0.0534 (14)	0.0610 (14)	0.0087 (11)	−0.0073 (11)	−0.0022 (11)
Cl2	0.0710 (4)	0.0760 (4)	0.0702 (4)	0.0197 (3)	0.0133 (3)	−0.0228 (3)
S2	0.0442 (3)	0.0460 (3)	0.0386 (2)	0.0026 (2)	0.0038 (2)	0.0006 (2)
C3	0.0747 (18)	0.0565 (16)	0.0842 (19)	0.0063 (13)	0.0069 (15)	−0.0203 (14)
Cl3	0.0469 (3)	0.0628 (4)	0.0668 (3)	−0.0073 (3)	−0.0014 (2)	0.0022 (3)
C4	0.0648 (16)	0.088 (2)	0.0586 (15)	−0.0091 (15)	0.0051 (12)	−0.0241 (14)
Cl4	0.0782 (4)	0.0568 (3)	0.0521 (3)	−0.0218 (3)	0.0002 (3)	−0.0077 (2)
C5	0.0681 (17)	0.089 (2)	0.0464 (13)	−0.0015 (15)	−0.0079 (12)	−0.0004 (13)
C6	0.0593 (14)	0.0587 (15)	0.0510 (12)	0.0058 (12)	−0.0032 (10)	0.0052 (11)
C7	0.0507 (12)	0.0482 (12)	0.0504 (12)	−0.0004 (10)	0.0129 (10)	−0.0050 (10)
C8	0.0484 (13)	0.0547 (13)	0.0649 (15)	0.0013 (11)	0.0063 (11)	0.0011 (11)
C9	0.0474 (14)	0.0686 (17)	0.104 (2)	0.0021 (13)	0.0106 (15)	−0.0012 (16)
C10	0.0620 (18)	0.080 (2)	0.117 (3)	−0.0077 (15)	0.0418 (19)	−0.0205 (19)
C11	0.096 (2)	0.110 (3)	0.078 (2)	−0.021 (2)	0.0527 (19)	−0.0195 (18)
C12	0.0713 (18)	0.104 (2)	0.0549 (15)	−0.0157 (16)	0.0211 (14)	−0.0103 (14)
C13	0.0543 (13)	0.0437 (11)	0.0488 (11)	0.0030 (10)	0.0013 (10)	−0.0025 (9)
C14	0.106 (2)	0.0571 (16)	0.0683 (16)	0.0105 (15)	0.0262 (16)	0.0091 (13)
C15	0.136 (3)	0.0542 (17)	0.087 (2)	0.0190 (18)	0.021 (2)	0.0141 (16)
C16	0.101 (2)	0.0524 (16)	0.101 (2)	0.0231 (16)	0.0052 (19)	−0.0041 (16)
C17	0.081 (2)	0.0691 (18)	0.093 (2)	0.0202 (16)	0.0250 (17)	−0.0065 (16)
C18	0.0625 (15)	0.0564 (14)	0.0685 (15)	0.0079 (12)	0.0151 (12)	0.0022 (12)
C19	0.0509 (12)	0.0489 (12)	0.0418 (10)	−0.0056 (10)	0.0038 (9)	−0.0051 (9)
C20	0.0661 (16)	0.0715 (17)	0.0694 (16)	0.0077 (14)	0.0185 (13)	0.0153 (14)
C21	0.0638 (16)	0.085 (2)	0.0807 (18)	0.0140 (15)	0.0132 (14)	0.0043 (16)
C22	0.0717 (17)	0.0748 (19)	0.0805 (18)	−0.0123 (15)	0.0323 (15)	−0.0246 (15)
C23	0.133 (3)	0.0698 (19)	0.089 (2)	0.001 (2)	0.067 (2)	0.0030 (17)
C24	0.104 (2)	0.0587 (16)	0.0691 (16)	0.0112 (16)	0.0391 (16)	0.0086 (13)
C25	0.0489 (12)	0.0529 (13)	0.0476 (11)	0.0001 (10)	0.0027 (9)	0.0010 (10)
C26	0.0633 (16)	0.0622 (16)	0.0788 (17)	−0.0062 (13)	−0.0140 (13)	0.0030 (14)
C27	0.0723 (19)	0.086 (2)	0.092 (2)	0.0028 (17)	−0.0307 (17)	0.0101 (18)
C28	0.089 (2)	0.0728 (19)	0.0721 (18)	0.0170 (17)	−0.0065 (16)	0.0129 (15)
C29	0.094 (2)	0.0549 (16)	0.084 (2)	0.0003 (15)	−0.0074 (17)	0.0117 (14)
C30	0.0720 (17)	0.0559 (15)	0.0745 (17)	−0.0037 (13)	−0.0139 (14)	0.0040 (13)
C31	0.0478 (11)	0.0440 (11)	0.0427 (10)	0.0032 (9)	0.0010 (9)	−0.0032 (9)
C32	0.0571 (14)	0.0646 (15)	0.0537 (13)	−0.0122 (12)	0.0037 (11)	−0.0048 (11)
C33	0.0673 (16)	0.0618 (16)	0.0674 (16)	−0.0072 (13)	−0.0089 (13)	−0.0123 (13)
C34	0.0823 (19)	0.0630 (16)	0.0556 (14)	0.0166 (14)	−0.0100 (13)	−0.0187 (13)
C35	0.0810 (19)	0.0830 (19)	0.0509 (13)	0.0202 (16)	0.0166 (13)	−0.0093 (13)
C36	0.0509 (13)	0.0642 (15)	0.0546 (13)	0.0048 (12)	0.0093 (10)	−0.0028 (11)

### Bis(triphenylsulfonium) tetrachloridocobaltate(II) (III) Geometric parameters (Å, º)

**Table:**

C1—C2	1.374 (3)	C16—H16	0.9300
C1—S2	1.787 (2)	C16—C17	1.372 (4)
C1—C6	1.374 (3)	C17—H17	0.9300
Cl1—Co1	2.2744 (6)	C17—C18	1.383 (4)
Co1—Cl2	2.2564 (7)	C18—H18	0.9300
Co1—Cl3	2.2893 (6)	C19—C20	1.374 (3)
Co1—Cl4	2.2761 (6)	C19—C24	1.362 (3)
S1—C19	1.791 (2)	C20—H20	0.9300
S1—C25	1.787 (2)	C20—C21	1.383 (4)
S1—C31	1.782 (2)	C21—H21	0.9300
C2—H2	0.9300	C21—C22	1.361 (4)
C2—C3	1.383 (3)	C22—H22	0.9300
S2—C7	1.790 (2)	C22—C23	1.346 (4)
S2—C13	1.788 (2)	C23—H23	0.9300
C3—H3	0.9300	C23—C24	1.386 (4)
C3—C4	1.375 (4)	C24—H24	0.9300
C4—H4	0.9300	C25—C26	1.371 (3)
C4—C5	1.367 (4)	C25—C30	1.381 (3)
C5—H5	0.9300	C26—H26	0.9300
C5—C6	1.380 (4)	C26—C27	1.381 (4)
C6—H6	0.9300	C27—H27	0.9300
C7—C8	1.372 (3)	C27—C28	1.355 (4)
C7—C12	1.376 (3)	C28—H28	0.9300
C8—H8	0.9300	C28—C29	1.362 (4)
C8—C9	1.384 (4)	C29—H29	0.9300
C9—H9	0.9300	C29—C30	1.375 (4)
C9—C10	1.361 (5)	C30—H30	0.9300
C10—H10	0.9300	C31—C32	1.383 (3)
C10—C11	1.363 (5)	C31—C36	1.371 (3)
C11—H11	0.9300	C32—H32	0.9300
C11—C12	1.390 (4)	C32—C33	1.380 (3)
C12—H12	0.9300	C33—H33	0.9300
C13—C14	1.379 (3)	C33—C34	1.363 (4)
C13—C18	1.378 (3)	C34—H34	0.9300
C14—H14	0.9300	C34—C35	1.374 (4)
C14—C15	1.389 (4)	C35—H35	0.9300
C15—H15	0.9300	C35—C36	1.385 (3)
C15—C16	1.368 (5)	C36—H36	0.9300
			
C2—C1—S2	114.01 (16)	C16—C17—H17	119.9
C6—C1—C2	121.8 (2)	C16—C17—C18	120.2 (3)
C6—C1—S2	124.09 (18)	C18—C17—H17	119.9
Cl1—Co1—Cl3	112.61 (3)	C13—C18—C17	119.0 (3)
Cl1—Co1—Cl4	107.41 (3)	C13—C18—H18	120.5
Cl2—Co1—Cl1	111.21 (3)	C17—C18—H18	120.5
Cl2—Co1—Cl3	104.92 (3)	C20—C19—S1	115.65 (18)
Cl2—Co1—Cl4	109.82 (3)	C24—C19—S1	123.5 (2)
Cl4—Co1—Cl3	110.89 (3)	C24—C19—C20	120.9 (2)
C25—S1—C19	104.97 (10)	C19—C20—H20	120.4
C31—S1—C19	104.61 (10)	C19—C20—C21	119.2 (3)
C31—S1—C25	103.74 (10)	C21—C20—H20	120.4
C1—C2—H2	120.7	C20—C21—H21	120.0
C1—C2—C3	118.6 (2)	C22—C21—C20	120.0 (3)
C3—C2—H2	120.7	C22—C21—H21	120.0
C1—S2—C7	101.57 (10)	C21—C22—H22	119.9
C1—S2—C13	106.37 (10)	C23—C22—C21	120.2 (3)
C13—S2—C7	106.36 (11)	C23—C22—H22	119.9
C2—C3—H3	120.0	C22—C23—H23	119.4
C4—C3—C2	119.9 (3)	C22—C23—C24	121.2 (3)
C4—C3—H3	120.0	C24—C23—H23	119.4
C3—C4—H4	119.7	C19—C24—C23	118.5 (3)
C5—C4—C3	120.7 (2)	C19—C24—H24	120.7
C5—C4—H4	119.7	C23—C24—H24	120.7
C4—C5—H5	119.9	C26—C25—S1	116.01 (19)
C4—C5—C6	120.2 (2)	C26—C25—C30	120.9 (2)
C6—C5—H5	119.9	C30—C25—S1	123.05 (18)
C1—C6—C5	118.7 (2)	C25—C26—H26	120.7
C1—C6—H6	120.7	C25—C26—C27	118.7 (3)
C5—C6—H6	120.7	C27—C26—H26	120.7
C8—C7—S2	115.83 (18)	C26—C27—H27	119.7
C8—C7—C12	121.9 (2)	C28—C27—C26	120.6 (3)
C12—C7—S2	122.2 (2)	C28—C27—H27	119.7
C7—C8—H8	120.6	C27—C28—H28	119.7
C7—C8—C9	118.8 (3)	C27—C28—C29	120.6 (3)
C9—C8—H8	120.6	C29—C28—H28	119.7
C8—C9—H9	120.0	C28—C29—H29	119.9
C10—C9—C8	120.0 (3)	C28—C29—C30	120.2 (3)
C10—C9—H9	120.0	C30—C29—H29	119.9
C9—C10—H10	119.5	C25—C30—H30	120.5
C9—C10—C11	120.9 (3)	C29—C30—C25	119.0 (3)
C11—C10—H10	119.6	C29—C30—H30	120.5
C10—C11—H11	119.8	C32—C31—S1	122.78 (17)
C10—C11—C12	120.5 (3)	C36—C31—S1	115.00 (17)
C12—C11—H11	119.8	C36—C31—C32	122.2 (2)
C7—C12—C11	118.0 (3)	C31—C32—H32	120.9
C7—C12—H12	121.0	C33—C32—C31	118.3 (2)
C11—C12—H12	121.0	C33—C32—H32	120.9
C14—C13—S2	123.44 (19)	C32—C33—H33	119.8
C18—C13—S2	115.20 (18)	C34—C33—C32	120.4 (3)
C18—C13—C14	121.3 (2)	C34—C33—H33	119.8
C13—C14—H14	120.7	C33—C34—H34	119.7
C13—C14—C15	118.6 (3)	C33—C34—C35	120.6 (2)
C15—C14—H14	120.7	C35—C34—H34	119.7
C14—C15—H15	119.8	C34—C35—H35	119.8
C16—C15—C14	120.4 (3)	C34—C35—C36	120.3 (3)
C16—C15—H15	119.8	C36—C35—H35	119.8
C15—C16—H16	119.8	C31—C36—C35	118.1 (2)
C15—C16—C17	120.4 (3)	C31—C36—H36	120.9
C17—C16—H16	119.8	C35—C36—H36	120.9
			
C1—C2—C3—C4	−1.2 (4)	C13—C14—C15—C16	0.7 (5)
C1—S2—C7—C8	−142.47 (18)	C14—C13—C18—C17	1.0 (4)
C1—S2—C7—C12	33.2 (2)	C14—C15—C16—C17	0.7 (6)
C1—S2—C13—C14	−74.6 (2)	C15—C16—C17—C18	−1.3 (5)
C1—S2—C13—C18	106.72 (19)	C16—C17—C18—C13	0.4 (5)
S1—C19—C20—C21	179.1 (2)	C18—C13—C14—C15	−1.6 (4)
S1—C19—C24—C23	−179.3 (2)	C19—S1—C25—C26	−105.1 (2)
S1—C25—C26—C27	−179.2 (3)	C19—S1—C25—C30	75.6 (2)
S1—C25—C30—C29	179.7 (2)	C19—S1—C31—C32	−27.9 (2)
S1—C31—C32—C33	−176.92 (19)	C19—S1—C31—C36	153.63 (18)
S1—C31—C36—C35	176.65 (19)	C19—C20—C21—C22	1.1 (5)
C2—C1—S2—C7	79.9 (2)	C20—C19—C24—C23	1.1 (4)
C2—C1—S2—C13	−169.06 (18)	C20—C21—C22—C23	−0.6 (5)
C2—C1—C6—C5	−2.2 (4)	C21—C22—C23—C24	0.5 (5)
C2—C3—C4—C5	−1.1 (5)	C22—C23—C24—C19	−0.7 (5)
S2—C1—C2—C3	−174.2 (2)	C24—C19—C20—C21	−1.3 (4)
S2—C1—C6—C5	174.7 (2)	C25—S1—C19—C20	172.01 (19)
S2—C7—C8—C9	177.1 (2)	C25—S1—C19—C24	−7.6 (2)
S2—C7—C12—C11	−176.9 (2)	C25—S1—C31—C32	81.9 (2)
S2—C13—C14—C15	179.8 (2)	C25—S1—C31—C36	−96.58 (19)
S2—C13—C18—C17	179.8 (2)	C25—C26—C27—C28	−0.2 (5)
C3—C4—C5—C6	1.8 (4)	C26—C25—C30—C29	0.4 (4)
C4—C5—C6—C1	−0.2 (4)	C26—C27—C28—C29	−0.2 (6)
C6—C1—C2—C3	2.9 (4)	C27—C28—C29—C30	0.8 (6)
C6—C1—S2—C7	−97.2 (2)	C28—C29—C30—C25	−0.8 (5)
C6—C1—S2—C13	13.9 (2)	C30—C25—C26—C27	0.1 (4)
C7—S2—C13—C14	33.1 (3)	C31—S1—C19—C20	−79.1 (2)
C7—S2—C13—C18	−145.57 (19)	C31—S1—C19—C24	101.3 (2)
C7—C8—C9—C10	−0.6 (4)	C31—S1—C25—C26	145.4 (2)
C8—C7—C12—C11	−1.4 (4)	C31—S1—C25—C30	−33.9 (2)
C8—C9—C10—C11	−0.1 (5)	C31—C32—C33—C34	0.3 (4)
C9—C10—C11—C12	0.0 (5)	C32—C31—C36—C35	−1.9 (4)
C10—C11—C12—C7	0.7 (5)	C32—C33—C34—C35	−1.7 (4)
C12—C7—C8—C9	1.4 (4)	C33—C34—C35—C36	1.3 (4)
C13—S2—C7—C8	106.44 (19)	C34—C35—C36—C31	0.5 (4)
C13—S2—C7—C12	−77.9 (2)	C36—C31—C32—C33	1.5 (4)

### Bis(triphenylsulfonium) tetrachloridocobaltate(II) (III) Hydrogen-bond geometry (Å, º)

**Table:**

*D*—H···*A*	*D*—H	H···*A*	*D*···*A*	*D*—H···*A*
C5—H5···Cl1^i^	0.93	2.85	3.632 (3)	142
C5—H5···Cl4^i^	0.93	2.92	3.672 (2)	139
C6—H6···C14	0.93	2.75	3.409 (4)	129
C8—H8···Cl4	0.93	2.82	3.633 (3)	147
C11—H11···Cl1^ii^	0.93	2.70	3.581 (3)	158
C12—H12···C6	0.93	2.88	3.547 (4)	130
C14—H14···C12	0.93	2.74	3.425 (4)	131
C18—H18···Cl3	0.93	2.68	3.525 (3)	152
C20—H20···Cl3	0.93	2.70	3.582 (3)	158
C23—H23···Cl4^iii^	0.93	2.91	3.519 (3)	124
C24—H24···C30	0.93	2.72	3.381 (4)	129
C26—H26···Cl1	0.93	2.81	3.649 (3)	151
C30—H30···C32	0.93	2.70	3.392 (4)	132
C32—H32···C4^iv^	0.93	2.78	3.564 (4)	142
C32—H32···C24	0.93	3.05	3.700 (4)	129
C35—H35···Cl2^v^	0.93	2.74	3.585 (3)	151

Symmetry codes: (i) *x*−1/2, −*y*+3/2, *z*−1/2; (ii) *x*+1/2, −*y*+3/2, *z*−1/2; (iii) −*x*+1/2, *y*−1/2, −*z*+3/2; (iv) −*x*, −*y*+1, −*z*+1; (v) −*x*+1, −*y*+1, −*z*+1.
